# RES complex is associated with intron definition and required for zebrafish early embryogenesis

**DOI:** 10.1371/journal.pgen.1007473

**Published:** 2018-07-03

**Authors:** Juan Pablo Fernandez, Miguel Angel Moreno-Mateos, Andre Gohr, Liyun Miao, Shun Hang Chan, Manuel Irimia, Antonio J. Giraldez

**Affiliations:** 1 Department of Genetics, Yale University School of Medicine, New Haven, CT, United States of America; 2 Centre for Genomic Regulation (CRG), Barcelona Institute of Science and Technology (BIST); Universitat Pompeu Fabra (UPF), Barcelona, Spain; 3 Yale Stem Cell Center, Yale University School of Medicine, New Haven, CT, United States of America; 4 Yale Cancer Center, Yale University School of Medicine, New Haven, CT, United States of America; University of Wisconsin—Madison, UNITED STATES

## Abstract

Pre-mRNA splicing is a critical step of gene expression in eukaryotes. Transcriptome-wide splicing patterns are complex and primarily regulated by a diverse set of recognition elements and associated RNA-binding proteins. The retention and splicing (RES) complex is formed by three different proteins (Bud13p, Pml1p and Snu17p) and is involved in splicing in yeast. However, the importance of the RES complex for vertebrate splicing, the intronic features associated with its activity, and its role in development are unknown. In this study, we have generated loss-of-function mutants for the three components of the RES complex in zebrafish and showed that they are required during early development. The mutants showed a marked neural phenotype with increased cell death in the brain and a decrease in differentiated neurons. Transcriptomic analysis of *bud13*, *snip1* (*pml1*) and *rbmx2* (*snu17*) mutants revealed a global defect in intron splicing, with strong mis-splicing of a subset of introns. We found these RES-dependent introns were short, rich in GC and flanked by GC depleted exons, all of which are features associated with intron definition. Using these features, we developed and validated a predictive model that classifies RES dependent introns. Altogether, our study uncovers the essential role of the RES complex during vertebrate development and provides new insights into its function during splicing.

## Author summary

RES complex is essential for splicing in yeast but its function and role during vertebrate development are unknown. Here, we combined genetic loss-of-function mutants with transcriptomic analysis and found that a subset of introns is particularly affected in RES complex knock-out background. Those introns display the major hallmarks of splicing through intron definition mechanisms (short introns, rich in GC and flanked by GC depleted exons). Moreover, *bud13*, *rbmx2* and *snip1* mutant embryos showed a marked brain phenotype with a RES-dependent introns enrichment in genes with neurodevelopmental function. Altogether, our study unveils the fundamental role of RES complex during zebrafish embryogenesis and provides new insights into its molecular function in splicing.

## Introduction

Splicing is critical step in eukaryotic gene expression and is an important source of transcriptomic complexity [1]. Splicing is carried out by the spliceosome, a large macromolecular complex that includes five small nuclear ribonucleoproteins (snRNPs; U1, U2, U4, U5 and U6) and hundreds of core and accessory proteins that ensure the accurate removal of introns from pre-mRNAs [2]. Canonically spliced introns are removed through two transesterification reactions during a complex process involving the recruitment and release of multiple core splicing factors [3]. However, many introns are recognized by different mechanisms depending on their specific features. Short introns with high GC content are believed to be spliced through an “intron definition” mechanism, in which initial U1-U2 pairing occurs across the intron. On the other hand, long introns surrounding short exons are recognized and spliced through “exon definition” mechanisms, in which the initial pairing bridges across the exon [4]. While these mechanisms are widely accepted, little is known about the specific factors associated with each process.

The pre-mRNA REtention and Splicing (RES) complex is a spliceosomal complex conserved from yeast to human. It is organized around the U2 snRNP-associated protein Snu17p/Ist3p (RBMX2 in human), which binds to both the pre-mRNA-leakage protein 1 (Pml1p; SNIP1 in human) and bud site-selection protein 13 (Bud13p; BUD13 in human) [5–7]. Snu17p interacts directly with the pre-mRNA and with Bud13p, and both proteins have been involved in splicing; on the other hand, Pml1p has been mainly linked to the retention of unspliced pre-mRNA in the nucleus [6–9]. Furthermore, the components of the RES complex cooperatively increase the stability and the binding affinity of the complex for the pre-mRNA [10–13], highlighting the importance of cooperative folding and binding in the functional organization of the spliceosome [10–12].

In yeast, the RES complex interacts with the 3’end of the intron in the actin pre-mRNA and is required for the first catalytic step of splicing [7,13]. Additionally, microarray-based studies have shown a global effect of the RES complex on yeast splicing [14,15]. Mutations in the RES-complex genetically interact with other spliceosomal components, and several introns seem particularly sensitive to RES complex loss-of-function, often in association with weaker splice sites [6,9,13,16–18]. Interestingly, disruption of the genes encoding for the three subunits of the RES complex show consistent phenotypes including slow growth, thermosensitivity [6] and alteration in budding pattern [19].

While the RES complex was identified in yeast, its function in vertebrates, the features recognized by this complex and its role during development are unknown. Here, we found that expression of the RES complex is enriched in the CNS during early development. We generated loss-of-function mutants for the three components of the RES complex in zebrafish using an optimized CRISPR-Cas9 gene editing system [20]. The three mutants showed severe brain defects with a significant decrease in the number of differentiated neurons and increased cell death in the brain and the spinal cord. We observed a mild retention across most introns, consistent with a global effect on splicing. However, a subset of introns was strongly affected. Importantly, these retained introns showed the hallmarks of intron definition [4,21], as they: (i) were shorter, (ii) had a higher GC content, and (iii) were neighbored by lower GC content exons. We developed a logistic regression model with these and other genomic characteristics that allowed us to discriminate between RES-dependent and independent introns with high accuracy. Altogether, these results provide new insights into the function of the RES complex and identify the features associated with RES-dependent splicing.

## Results

### RES complex is essential for zebrafish early embryogenesis

**Fig 1:**
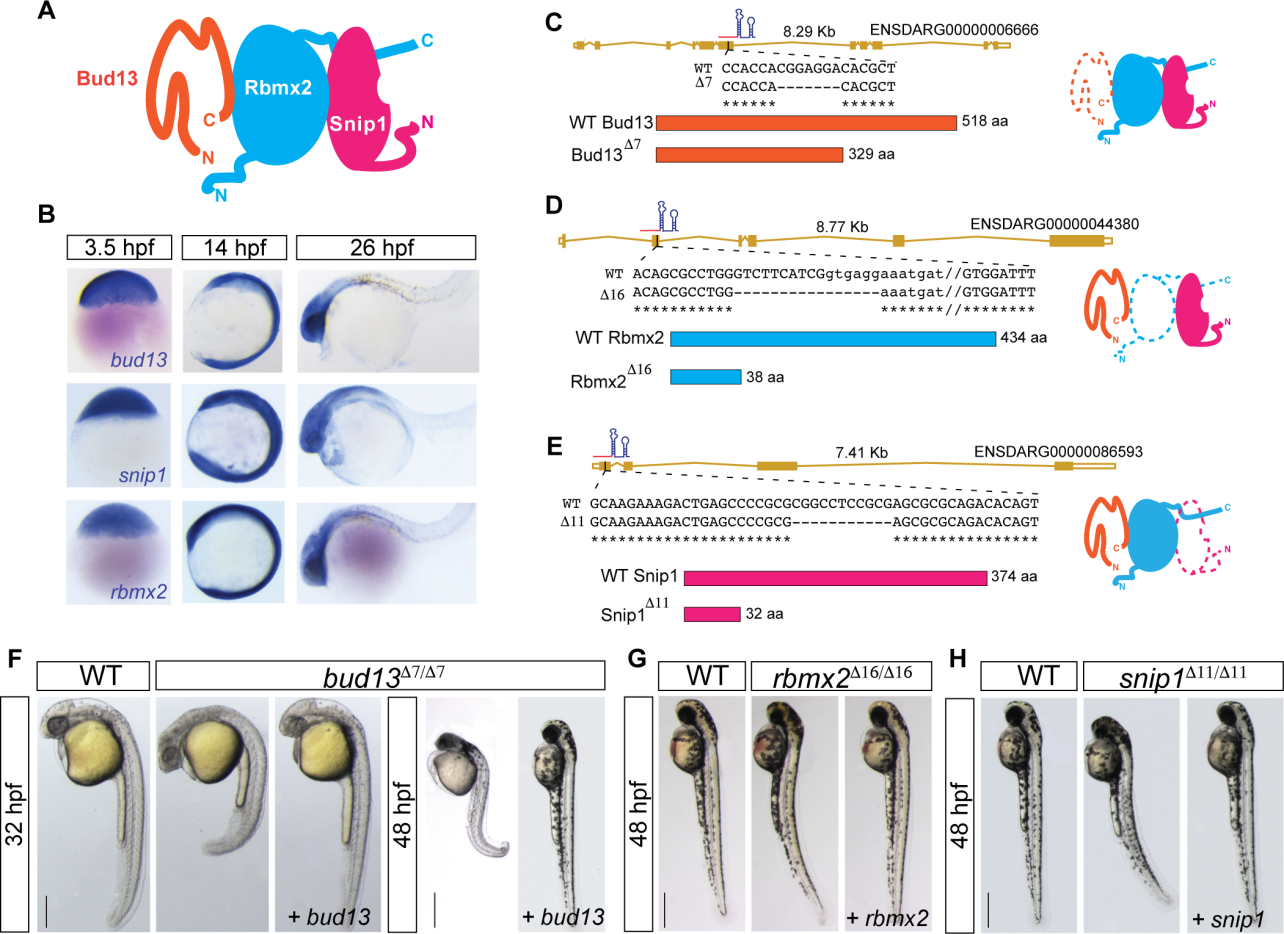
RES complex is essential for early vertebrate development. **(A)** Schematic model of the RES complex adapted from Brooks et al. [8]. Rbmx2 (light blue) is the core subunit with an RRM-domain structure. Bud13 (orange) and Snip1 (pink) interact with Rbmx2 (light blue). **(B)** In situ hybridization showing spatial and temporal expression of RES complex members. **(C-E)** Gene models of the mutant allele generated using CRISPR-Cas9-nanos. **(C)** *bud13*, 7 nt deletion in exon 6 generated a premature stop codon. **(D)** *rbmx2*, 16 nt deletion removed exon-intron boundary at exon 2 (exon capital letter, intron lower letter). **(E)** *snip1*, 11 nt deletion in exon 1 generated a premature stop codon. f-g) Lateral view of RES complex mutant embryos, their corresponding WT sibling and mutants injected with the cognate mRNA. **(F)** *bud13* mutant at 32 hpf (scale bar: 0.35mm) and at 48 hpf (scale bar: 0.5mm). **(G, H)** *rbmx2* and *snip1* mutant at 48 hpf respectively. WT: represent phenotypically wild type sibling from the same mutant fish line.

To determine the role of the RES complex during vertebrate development, we first analyzed its expression pattern during development. *bud13*, *rbmx2* and *snip1* (Fig 1A) are maternally expressed (Fig 1B, S1D and S1F Fig), and later in development their mRNAs are strongly expressed in the central nervous system (CNS) (26 hours post fertilization, hpf), (Fig 1B) suggesting that RES complex may be required for brain development. Next, we generated mutant zebrafish lines for *rbmx2*, *snip1* and *bud13* using an optimized CRISPR-Cas9 system (S1A Fig) [20]. We identified a seven-nucleotide deletion in *bud13* (*bud13*^*∆7/∆7*^), a sixteen-nucleotide deletion in *rbmx2* (*rbmx2*^*∆16/∆16*^) and eleven-nucleotide deletion in *snip1* (*snip1*^*∆11/∆11*^). These mutations are predicted to cause premature stop codons and disrupt protein function (Fig 1C–1E). Zygotic mutants for the three components of the RES complex showed a Mendelian ratio of homozygous mutant embryos. We observed strong structural brain defects and widespread cell death in the CNS at ~30 hpf in *bud13* and ~48 hpf in *rbmx2* and *snip1* (Fig 1F–1H; S1B, S1C, S1E and S2B Figs). The embryo progressively degenerates and mutants die by 4–5 dpf. Earlier depletion of *bud13* gene expression using morpholino antisense oligonucleotide targeting the AUG start site showed a more severe phenotype affecting not only the brain and CNS but also other tissues (e.g. mesoderm) (S2A Fig), consistent with a repression of the maternal contribution in the morphants compared to the zygotic mutants. Both, the mutant and morphant phenotypes were specific and fully rescued by injection of the cognate mRNA (*bud13*, *rbmx2 or snip1*) (Fig 1F–1H) or human mRNA (h*BUD13*) up to 6 dpf (S2B Fig). Interestingly, rescuing with lower amounts of h*BUD13* mRNA partially phenocopied the *rbmx2* and *snip1* mutant phenotypes at 48 hpf (S2C Fig). These results suggest that the onset of the zygotic phenotype in *rbmx2* and *snip1* mutants (48 hpf) is likely due to differences in the maternal mRNA contribution and/or protein stabilities for these genes. Taken together, these results demonstrate that (i) the mutant phenotypes are specific to the targeted loci, (ii) the RES complex is essential for embryonic development and suggest that (iii) the biochemical function of Bud13, and presumably of the RES complex, may be conserved from human to zebrafish.

**Fig 2:**
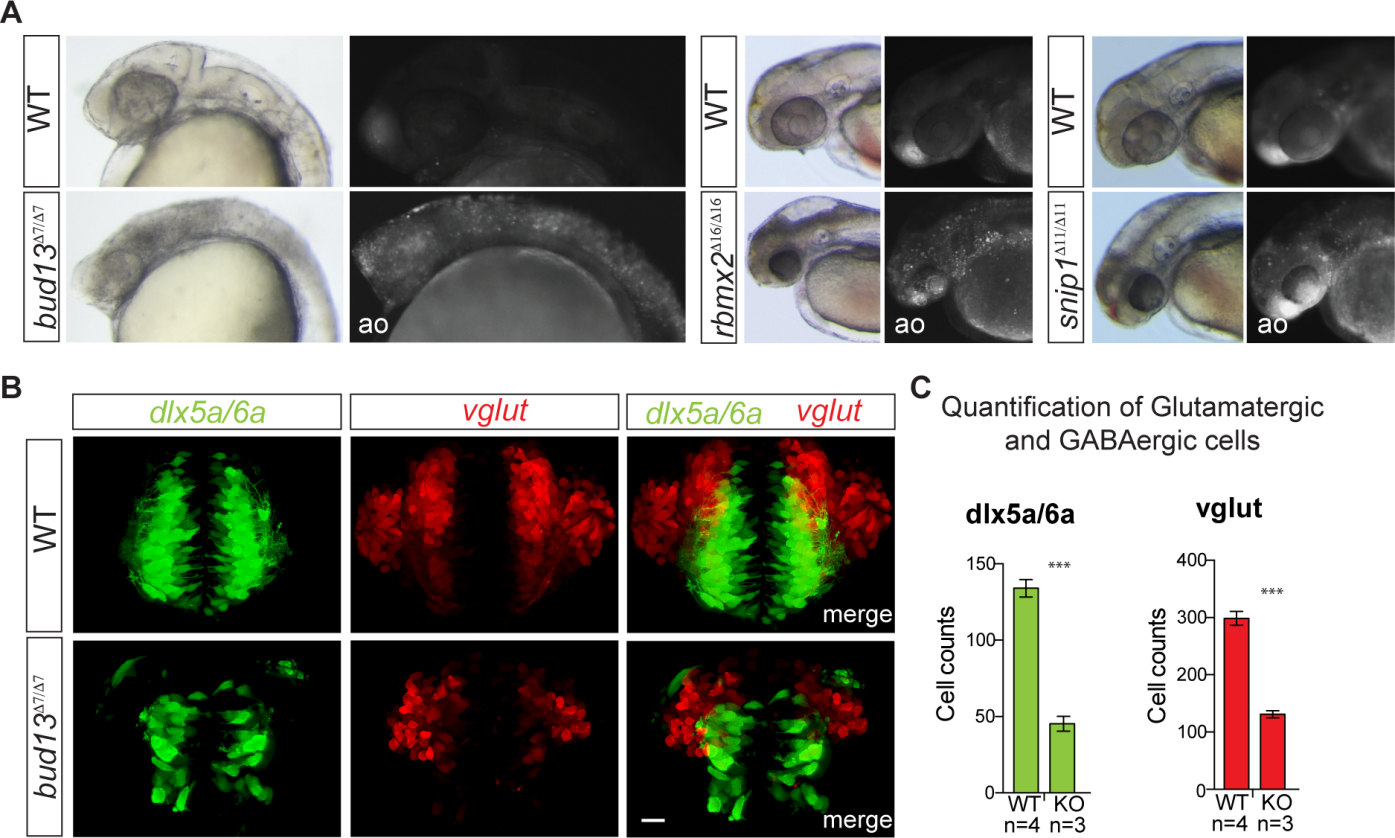
RES complex is required during zebrafish brain development. **(A)** Acridine orange (ao) staining of zebrafish mutant embryos for *bud13* (30–32 hpf), *rbmx2* and *snip1* (48 hpf). Mutants show a marked degree of cells with nuclear uptake of ao compared to WT sibling most predominantly in the head. WT: represent phenotypically wild type sibling from the same mutant fish line. **(B)** Maximum intensity projections of individual and merged channels (GFP and dsRed) of 3D confocal images of *bud13*^Δ7/Δ7^ and their wild-type siblings (scale bar 20 μm) in transgenic lines that label GABAergic neurons and precursors (Tg[dlx6a-1.4kbdlx5a/ dlx6a:GFP]) and glutamatergic neurons (Tg[vglut:DsRed]). WT: represent phenotypically wild type sibling from the same mutant fish line. **(C)** Total number of dlx5a/6a:GFP+ cells (GABAergic neurons and precursors) and vglut:DsRed+ cells (glutamatergic neurons) in the forebrain of the *bud13*^Δ7/Δ7^ (n = 3) and WT sibling (n = 4) were quantified. *** vglut: *P* = 2 x 10^−4^; ***dlx5a/6a: *P* = 3 x 10^−4^ (one-way ANOVA).

To determine the role of the RES-complex in splicing and gene expression, we analyzed polyA+ RNA from each mutant at the onset of the phenotype. As a control, we analyzed the transcriptome of stage-matched wild type siblings (See material and methods). We observed 621 up-regulated genes in all three mutants, that were significantly enriched for functions related to i) cell death (e.g. *p53*, *casp8* and *puma*) (S4D Fig), consistent with the appearance of apoptosis in the brain (Fig 2A and S1B Fig), and ii) spliceosomal components, suggesting a compensatory effect upon a general splicing deficiency (See Materials and Methods for details, S4A, S4C Fig and S1 Table). In contrast, 745 genes were consistently down-regulated and were enriched for genes involved in transcriptional regulation (such as *sox19b*, *atoh7*, *pou3f1*) and nervous system development (e.g. *neurod1/4/6b*, *sox1a*) (S4B Fig and S1 Table).

Importantly, *bud13*^*∆7/∆7*^ mutants showed normal neural induction, morphogenesis and regionalization. For example, key brain areas such as the *zona limitans intrathalamica* (ZLI; dorsal *shh* in the diencephalon), the mid/hind-brain boundary (*pax2a* expression), and rhombomeres 3 and 5 (*krox20* expression) were properly specified (S3 Fig). This suggests that the zygotic function of *bud13* is not required for initiation of neural linage patterning and specification. In contrast, we observed a reduction in the number of differentiated neurons, including both excitatory and inhibitory neuronal populations, with significant decrease in glutamatergic as well as GABAergic neurons in the forebrain in *bud13*^*∆7/∆7*^ embryos at 32 hpf (Fig 2B and 2C). These results suggest that zygotic RES activity is required for neuronal differentiation and/or survival, but not for neural induction and early brain patterning.

### RES complex mutants show widespread intron mis-splicing

**Fig 3:**
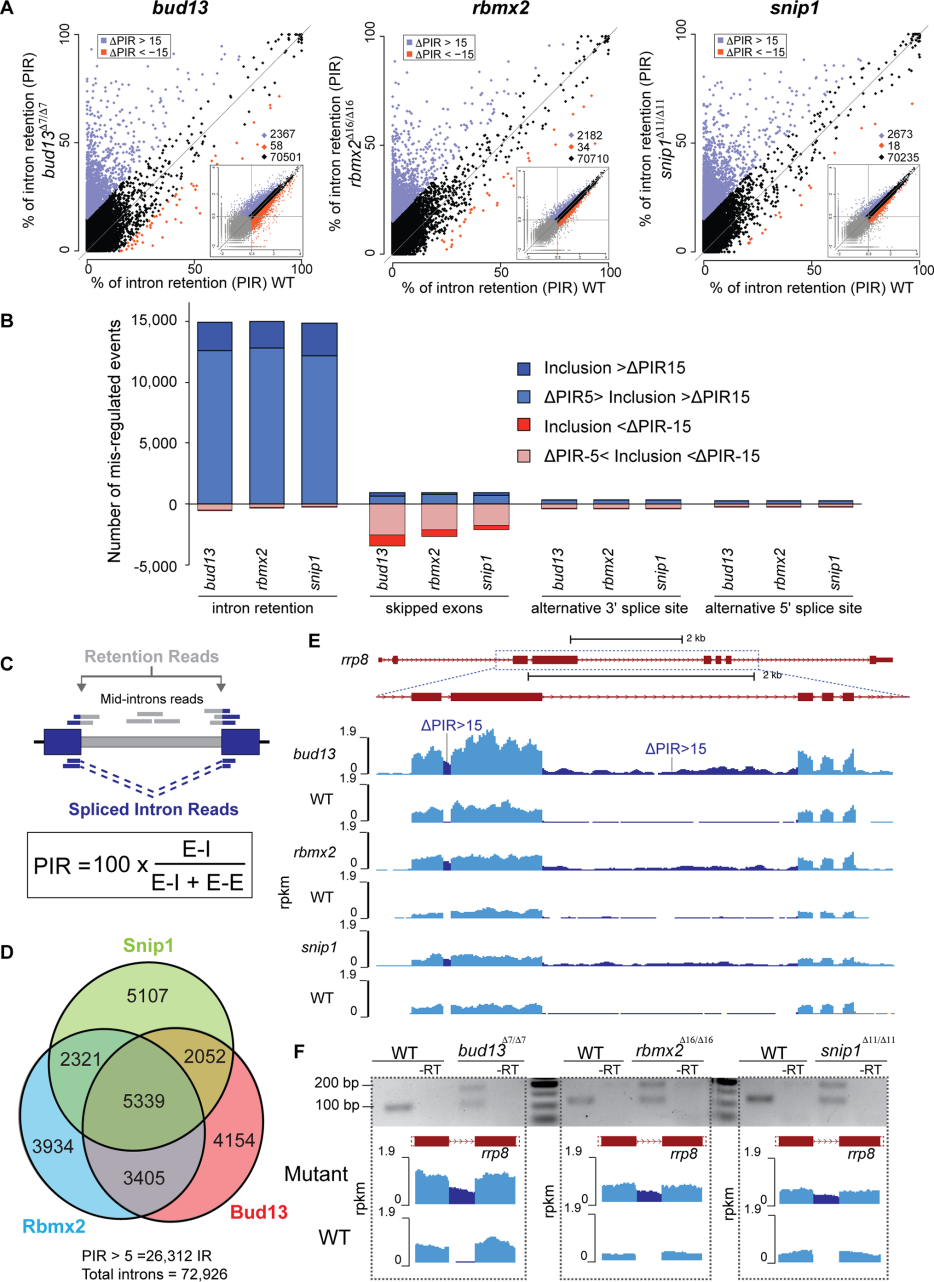
RES complex mutants show mild widespread intron mis-splicing. **(A)** Biplot illustrating percent intron retention (PIR) in each of the RES mutants and the corresponding phenotypically wild type (WT) siblings. Blue and red dots correspond to introns with higher inclusion in the mutant and WT, respectively, using a cutoff of ∆PIR >15. Insets show exon gene expression levels in the same conditions (see S4 Fig for details). **(B)** Stacked barplot showing the number of mis-regulated events upon RES loss-of-function. Clearly, most changes are intron retention supporting the role of the RES complex in splicing. **(C)** Scheme showing how PIR was measured (adapted from Braunschweig et al. [22]; see Methods for details). **(D)** Euler diagram showing the number of retained introns (∆PIR>5) in the three mutants and the inter-mutant overlaps. **(E)** RNA-seq read density across the *rrp8* gene in *bud13*, *rbmx2* and *snip1* mutants and their corresponding phenotypically WT siblings. Intronic signal increases in RES mutants (∆PIR>15) (dark blue) (dotted square box). **(F)** RT-PCR assays validate the increased retention of an *rrp8* intron (from panel E) in *bud13*, *rbmx2* and *snip1* mutants compared to the corresponding phenotypically WT siblings.

To determine the global impact of the RES complex mutants on splicing, we analyzed the level of intron retention (IR) across the transcriptome. Briefly, for any given intron, the percent intron retention (PIR) is calculated as the average number of reads mapping to the 5’ and 3’ Exon-Intron (E-I) junctions over the average number of reads mapping E-I junctions plus any Exon-Exon (E-E) junction that supports removal of that given intron (Fig 3C) [22]. We found that 74–79% of introns showed increased retention (ΔPIR > 0) in the individual mutants compared to wild-type siblings (72,926 introns, with sufficient coverage, see Methods for details). In contrast, a significantly smaller set of introns (7–9%) showed decreased retention in the mutants (Fig 3A and S7C Fig, *P*<2.2e-16, Fisher exact test). Other types of splicing events were less impacted upon RES depletion (Fig 3B), although a substantial number of exons became skipped (S7D Fig) in the loss-of-function mutants, consistent with a general disruption of splicing. Interestingly, the three mutants shared 5,339 (~35%) introns with a medium-high level of retention (ΔPIR>5) (Fig 3D), consistent with a common function in the RES complex. This is likely an underestimate because each mutant was analyzed at the onset of the mutant phenotype, which is different across these mutants likely due to different level of maternal recue or protein stability. We observed mild widespread intron retention, yet ~3.5% of introns showed strong increased retention across each mutant (ΔPIR >15, S7C Fig), suggesting that a subset of introns have a stronger dependence for RES function *in vivo*. This effect was validated by RT-PCR for a subset of candidates for each mutant (Fig 3E, Fig 3F and S5 Fig), including *wdr26b* and *ptch2*, that when mutated in humans cause neurodevelopmental disorders [23,24]. Genes with strongly retained introns (ΔPIR >15) in at least two of the three mutants were enriched in transcriptional regulation and DNA binding (S6A Fig), including known regulators of vertebrate development and neuronal differentiation (e.g. *irx1a*, *smn1*, *enc1*, *smad4*, *tbx2a*, and *nkx6*.*1/6*.*2*; S2 Table). Further analyses revealed complex interactions between pre-mRNA splicing and mRNA abundance in response to RES complex depletion. Genes with at least one strongly retained intron (ΔPIR >15) had lower expression in the mutants compared to genes without intron retention (ΔPIR < 2) (S6B Fig; *P*<10–7 for the three mutants, Wilcoxon Sum Rank test). Conversely, genes that were differentially expressed in the three mutants (down- or up-regulated) show significantly higher retention (higher ΔPIR) in the mutants compared to their WT siblings (S6C Fig; *P*<10–8 for all comparisons in the three mutants, Wilcoxon Sum Rank test). Finally, genes with increased expression significantly overlapped with those with at least one strongly retained intron (ΔPIR>15) (S6D Fig). Taken together, our analyses indicate that the RES complex has a global effect on splicing, and is strongly required for a subset of introns in genes involved in transcriptional regulation and neural development.

### Distinguishing features and classes of retained introns in the RES complex

**Fig 4:**
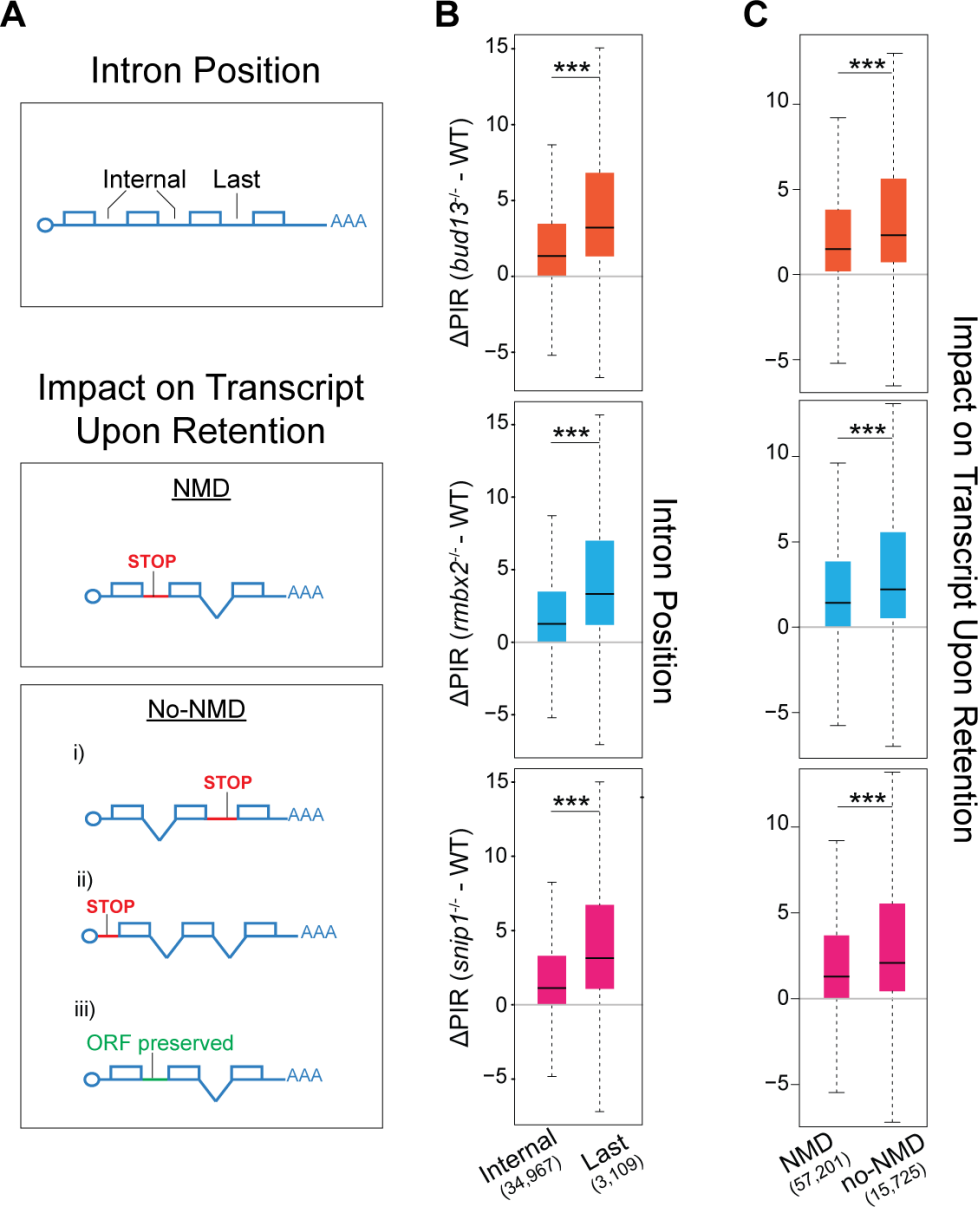
Genome-wide analysis of intron retention in RES complex mutants. **(A)** Schematic representation of intron features analyzed in (B) and (C). Top: Intron position. “Internal” introns correspond to all introns excluding the first two and last three introns. Bottom: NMD vs non-NMD triggering introns. Introns predicted to cause NMD upon retention introduce a premature termination codon (PTC) further than 50 nucleotides upstream of an exon-exon junction. Introns predicted not to cause NMD (noNMD) may correspond to: (i) last introns, (ii) introns in UTRs or non-coding genes, or (iii) introns that preserved the ORF upon retention (multiple of three nucleotides with no in-frame stop codons). **(B)** Box plots showing the ∆PIR of last and internal introns in the three different mutant of the RES complex. Only genes with more than 10 introns were considered for the analysis. *bud13* *** *P* = 4.37x10^-201^; *rbmx2* *** *P* = 2.98x10^-202^; *snip1* *** *P* = 4.76x10^-195^ (Wilcoxon rank sum test). **(C)** Box plots showing the ∆PIR of introns predicted to trigger nonsense mediated decay (NMD) upon retention and those predicted not to trigger NMD (no-NMD) in the three different mutant of the RES complex. *bud13* *** *P* = 5.83x10^-208^; *rbmx2* *** *P* = 4.94x10^-184^; *snip1* *** *P* = 1.74x10^-183^ (Wilcoxon rank sum test).

To identify features that are primarily associated with RES complex function, we first defined a set of introns that were confidently dependent on RES for proper splicing (1,409 “RESdep” introns with ∆PIR>15, ≥1.5-fold net increase in intron reads; see Methods) (Fig 3C). As a control set (Ctr), we defined 5,574 introns with ∆PIR<0.5 in all three mutants, and evaluated the enrichment of 44 features, many of which have been previously associated with intron retention [22] (S3 Table). Consistent with the genome-wide patterns (Fig 4), strongly retained introns were enriched for last introns (Fig 4A and 4B and S7A Fig) and introns that do not trigger NMD when mis-spliced (Fig 4A and 4C and S7B Fig), suggesting that their accumulation is in part likely due to reduced degradation of the unspliced transcript isoform. However, a significant fraction of highly retained introns was predicted to elicit NMD upon inclusion. We thus hypothesized that this subset of NMD-triggering introns contains specific features that would maximally associate with RES-dependent mechanisms. Based on this, we also separately analyzed introns that were predicted to trigger NMD (574) and those that were not (569) (see Methods).

**Fig 5:**
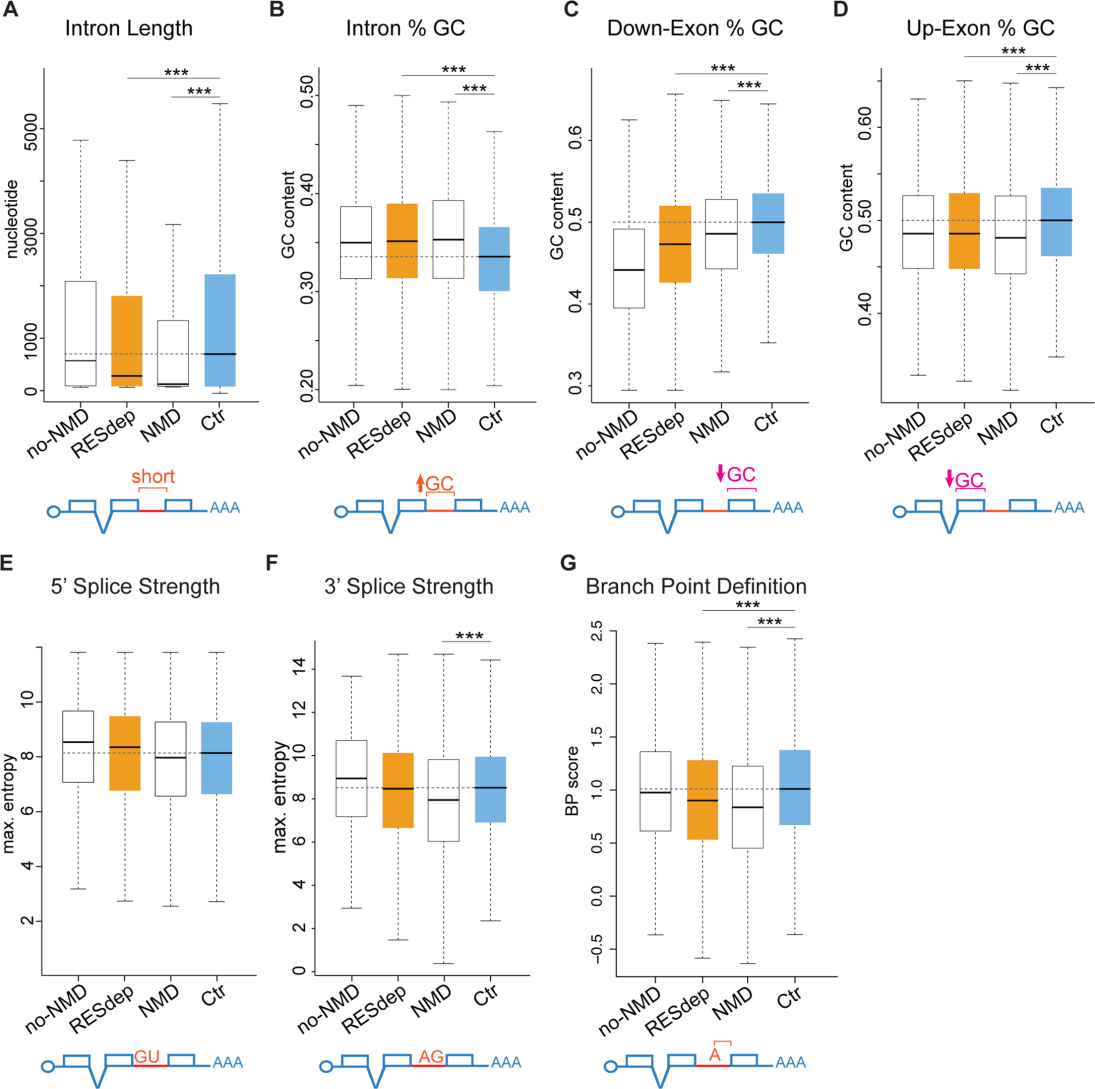
**Molecular features that determine intron retention in RES are associated with splicing through intron definition (A-G).** Box plots showing the median (black solid line) and the distribution of values for multiple intron-exon features of several groups of introns of interest. “RESdep”, all highly retained introns (∆PIR >15) in at least two out the three mutants in the RES complex; “no-NMD”, subset of “RESdep” retained introns that are predicted not to trigger NMD; “NMD”, subset of “RESdep” retained introns predicted to trigger NMD; and “Ctr”, control set of introns with a ∆PIR cutoff < 0.5 in the three RES complex mutants (see Materials and Methods for details). (*** *P* ≤ 0.001; Mann-Whitney-U test). Branch point (BP) definition: BP Score of best-predicted BP. Scored base on Corvelo et al. [24] (See Materials and Methods for details).

RES-dependent introns (i) were significantly shorter than the control set (Fig 5A, median of 281 nt versus 749 nt in the control, *P*<0.001, Mann-Whitney-U test), (ii) had elevated GC content (Fig 5B, *P*<0.001 Mann-Whitney-U test), (iii) were flanked by exons with lower GC content (Fig 5C and 5D, S8C and S8D Fig), and (iv) had weaker branch point (BP) consensus sequences [25,26] (Fig 5G and S8 Fig). Remarkably, at the genome-wide level, short introns and introns with high GC content also showed a higher retention across all three mutants compared to wild type siblings (S9 Fig). NMD-triggering introns further showed weaker core splicing signals, including acceptor (3’) and donor (5’) splice sites and BP consensus sequences than any other intron set (Fig 5E–5G), providing an explanation for their increased sensitivity upon RES complex disruption despite being subject to NMD.

**Fig 6:**
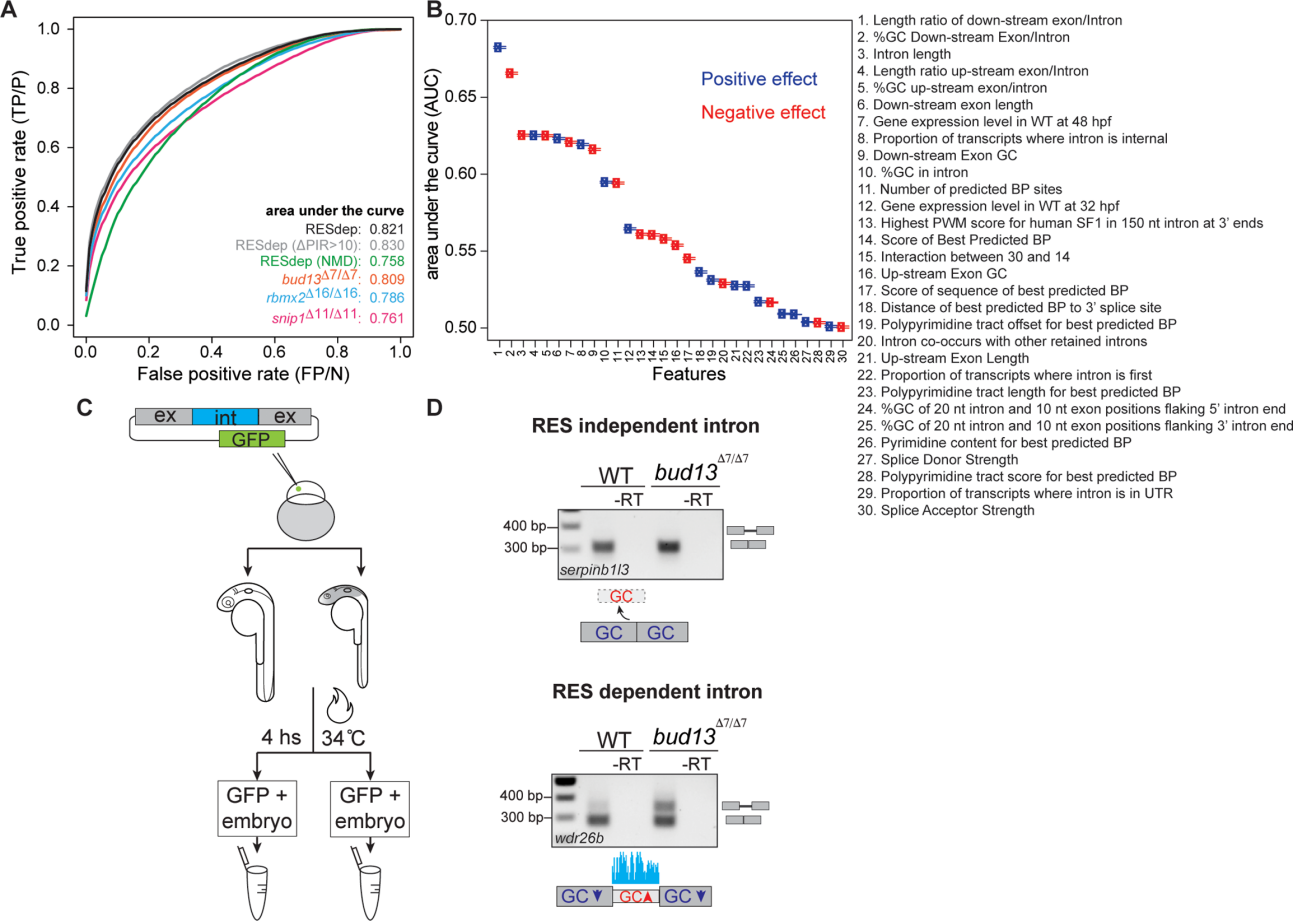
Logistic regression model can accurately classify RES-dependent and non-dependent introns. **(A)** Classification performance of logistic regression models for different data sets of differentially retained vs. Ctr introns. ROC curves are averaged over 10,000 repeated holdout experiments where models have been trained with randomly sampled subsets of 90% (1,268) of the RESdep introns versus 1,268 Ctr introns with 30 features (S3 Table) and Lasso feature selection. Classification performance was estimated using the remaining 10% (141) RESdep introns and 141 randomly sampled Ctr introns. Having held fixed parameters, the same model was used to estimate classification performance with randomly sampled 141 introns from the other RES-dependent data sets, namely: (i) “RESdep (∆PIR>10)” introns from the “RESdep” set with ∆PIR > 10 in all three mutants (871 introns); (ii) “RESdep (NMD)”, introns from the “RESdep” set predicted to trigger NMD when retained (574 introns); (iii) “*bud13*^*∆7/∆7*^”, introns with ∆PIR>15 upon *bud13* mutation at 32 hpf (2,363 introns); (iv) “*rbmx2*^*∆16/∆16*^”, introns with ∆PIR>15 upon *rbmx2* mutation at 48 hpf (2,186 introns); and (v) “*snip1*^*∆11/∆11*^”, introns with ∆PIR>15 upon *snip1* mutation at 48 hpf (2,675 introns). 95% confidence interval of reported average AUCs corresponds to AUC ± 0.001. **(B)** Capacity of each feature to discriminate between “RESdep” and “Ctr” introns, measured as AUC (average area under ROC curve) when used as the only feature in a one-feature logistic regression model. **(C)** Schematic of the experiment performed to validate the predicted features, see Material and Methods for details. **(D)** RES dependent but not RES independent intron was retained in *bud13*^*∆7/∆7*^ mutant as the regression model predicted. The validation experiment was done using two independent biological replicates (see S12A Fig).

Next, we assessed how genomic and transcript features define the dependence on the RES-complex. We applied a logistic regression model using 30 features as predictor variables (S3 Table), and defined a response variable classifying each intron as RES-dependent or non-dependent. We developed a model using 90% of the RESdep and a size-matched subset of control introns as training set, and validated the model on the remaining 10% of the data (see Material and Methods). We found a high performance in the classification of introns, with an average Area Under the ROC curve (AUC) of 0.821 (Fig 6A). This model was able to classify the impact of mutating each individual RES component with an AUC of ≥0.76. Subsequently, we analyzed the individual contribution of each feature to the model and their potential to reduce the null deviance (see Methods). Consistent with results in Fig 5, this analysis identified four important features i) the ratio between exon and intron length, ii) the ratio between exon and intron GC content, iii) gene expression levels and iv) the position of the intron within the transcript (last intron effect) (Fig 6B and S10 Fig).

To further test the model on an independent set of introns, we applied the model to 108,470 introns (S6 Table) without sufficient read coverage across our six RNA-seq samples and were not used for previous analyses. We then selected the top 100 introns predicted to be *bud13*-dependent and -independent and plotted their ∆PIR values based on RNA-seq data for the *bud13* mutant and the corresponding control (S11A Fig). The majority of predicted *bud13*-dependent introns were substantially retained, whereas introns that were predicted to be unaffected showed a median ∆PIR close to zero (0.91) (S11A Fig). The false prediction rate (FPR) is 0.34 for the *bud13* dependent introns and 0.16 for the unaffected introns, consistent with the AUC values reported above. Repeating this independent validation with *rbmx2* and *snip1* dependent introns (S11A Fig) showed FPRs of 0.33 and 0.03 for *rbmx2*, and 0.44 and 0.04 for *snip1*. The lower FPRs for the unaffected introns reflect that predicting unaffected introns can be obtained with higher performance, likely because the data contain a considerably larger amount of unaffected than affected introns. We further validated these predictions by RT-PCR assays for five predicted RES-dependent and five RES-independent introns (S11B Fig). Thus, altogether, our logistic regression analysis can identify introns dependent on the RES complex based on specific features within the genomic locus and the transcript.

Finally, to test our logistic regression model in isolation from the genomic context, we assayed four introns, two RES dependent and two RES independent, using minigene constructs including the tested intron plus the two flanking exons (Fig 6C and 6D and S12 Fig). Minigenes were cloned into a transgenesis vector (S12B Fig), and injected into 1-cell stage embryos. To assess the splicing pattern, we carried out a RT-PCR followed by PCR (S4 Table). From the subset of four different introns, three events were validated by the RT-PCR and gel electrophoresis as predicted by our model (Fig 6A, 6B, 6C and 6D and S12 Fig). These introns were in *serpinb1l3* and *col1a2* (predicted as RES independent) and *wdr26b* (predicted as RES dependent with ∆PIR>15) transcripts. Nevertheless, we detected no intron retention in *ptch2*, suggesting that its retention upon RES depletion may depend on its genomic context.

## Discussion

Splicing regulatory information is encoded by multiple sequence features, from the core signals (splice donor and acceptor and branch point) to other, less understood, sequence elements [27–29]. Our results identify intronic features that are associated with RES-dependent splicing across the transcriptome. These features can be used to discriminate a large fraction of RES-dependent from independent introns. Although loss-of-function for RES complex components caused mild intron retention across the transcriptome, we observed a subset of introns that were strongly accumulated across mutants of the RES complex. Recent *in vitro* studies on the single intron of the actin gene in yeast showed that the RES complex binds at the 3’ of the intron, between the BP and the acceptor site [12]. We observed that introns that more strongly depend on the RES complex show weaker BP consensus sequences. Furthermore, this subset of introns were shorter and had higher GC content, an association that is particularly striking in zebrafish, since short introns normally have lower GC content [21]. RES-dependent introns are flanked by exons with a lower GC content than RES-independent introns (Fig 5, S9C and S9D Fig). Therefore, these observations are consistent with a model whereby RES-dependent introns are mainly spliced through intron definition [4,21]. This association is surprising, since biochemical evidence suggest that the RES complex joins the spliceosome after recognition of the splice sites [12], and that the RES complex is not needed for spliceosome assembly *in vitro* but for U1 and U4 snRNP dissociation before the first catalytic step [7]. One possible explanation for this apparent discrepancy is that RES complex components play a role in early splice site recognition *in vivo* and therefore that the biochemical functions reported in a limited set of RNAs reflect limitations of *in vitro* splicing reactions. Alternatively, the RES complex may not be involved in early splice site recognition, but could be a limiting factor for splice site pairing or other steps in spliceosome assembly progression *in vivo*, particularly for introns defined by intron definition, highlighting differences in molecular pathways for intron- and exon-defined splicing. These concepts are in line with increasing evidence that splice site selection can be modulated at late stages of spliceosome assembly or even catalysis [14,27,30–35]. Finally, some RES-dependent introns also have weaker donor and acceptor splice site consensus sequences, and thus are expected to be more sensitive to defects on the splicing machinery. This is consistent with previous studies in yeast, which found that weaker 5’ splice sites increased susceptibility to RES loss-of-function [6].

An unexpected observation from genome-wide analyses of core splicing factor loss-of-function experiments is that each factor seems to differentially affect a specific subset of introns and exons [32,36]. This suggests that splicing of each intron in the genome is limited by specific core factors, depending on its combination of sequence features, as we observed for RES-dependent introns. As such, disruption of core splicing factors is predicted to produce unique phenotypes dictated by its expression, and the expression and function of genes that contain the subsets of introns sensitive to that factor. Consistent with this hypothesis, RES complex is required during brain development and neuronal survival, and mis-regulated introns are found in genes with well-known functions in neurodevelopment (e.g. *irx1a*, *smn1*, *enc1*, *smad4*, *tbx2a*, and *nkx6*.*1/6*.*2*). Specifically, zygotic mutants in *bud13*, *snip1* or *rbmx2* show microcephaly and decreased populations of GABAergic and glutamatergic neurons, despite normal specification and regionalization of the CNS (Fig 1, Fig 2 and S3 Fig). This phenotype is different from those described for a few other spliceosomal-related mutants in zebrafish [37–40]. For instance, while *sf3b1* is required for early neural crest development [40], loss of another core component of the spliceosome, *prpf8*, results in massive neuronal cell death and impaired myeloid differentiation [37]. These differences might be caused by the different half-life of the maternal proteins in the zygotic mutants. Alternatively, different components of the splicing machinery might be essential in a cell-type/tissue specific manner during early development. This may also explain why mutations in specific spliceosomal components cause human diseases with diverse phenotypes, such as Taybi-Linder syndrome, microcephalic osteodysplastic primordial dwarfism type I and retinitis pigmentosa [41–43]. Interestingly, a mutation in human *SNIP1* (p.Glu366Gly) has been associated with epilepsy and skull dysplasia [44]. Our data shows that human *BUD13* can rescue loss of *bud13* function in zebrafish, and future studies will be needed to determine whether Bud13 has a conserved function during brain development in humans (S2B and S2C Fig).

**Fig 7:**
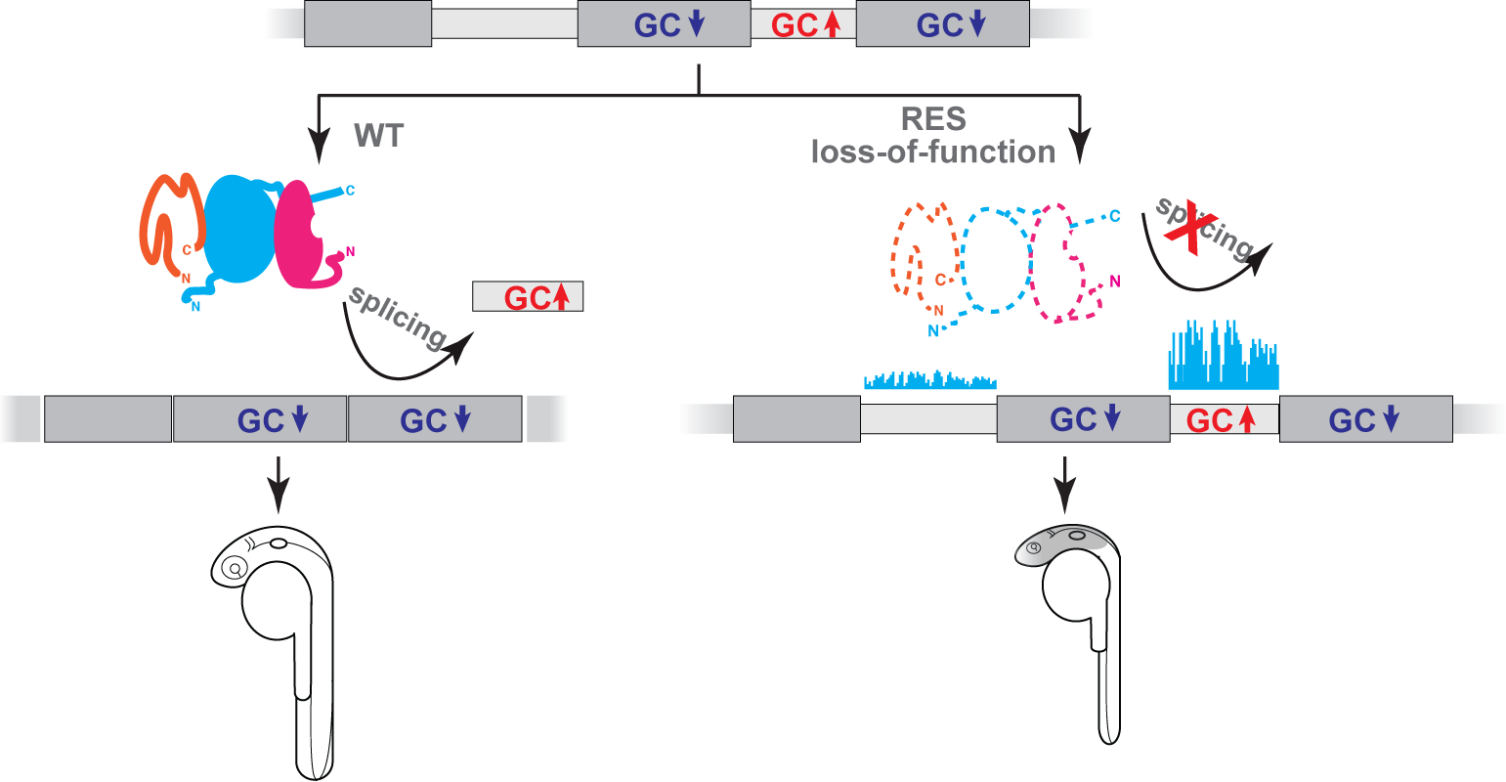
Schematic integrative model of the RES complex function in splicing. The loss-function of the RES complex induces a global weak defect in splicing, but a strong retention of a subset of introns with the following features: these introns are short, have a higher GC content and flanked by GC-depleted exons, features associated with intron definition splicing mechanism. This splicing defect leads to a brain-related phenotype.

In summary, we have shown that RES complex disruption in zebrafish hinders splicing, but is not essential for the removal of most introns, indicating that such introns can be efficiently defined and spliced through RES-independent mechanisms. However, we found that a subset of introns is particularly affected by RES complex removal and that those introns display the major hallmarks of splicing through intron definition mechanisms. From a functional perspective, RES-dependent introns are in genes enriched for transcription factors and neurodevelopmental regulatory functions, thus resulting in brain developmental defects in loss-of-function zygotic mutants (Fig 7). Future studies will be needed to understand how spliceosomal mutations disrupt splicing of different genes by affecting specific limiting steps in pre-mRNA splicing resulting in diverse disease phenotypes.

## Materials and methods

### Ethics statement

Fish lines were maintained in accordance with research guidelines of the International Association for Assessment and Accreditation of Laboratory Animal Care, under a protocol approved by the Yale University Institutional Animal Care and Use Committee (IACUC).

### Zebrafish maintenance, mating and embryos image acquisition

Wild-type zebrafish embryos were obtained through natural mating of TU-AB and TLF strains of mixed ages (5–17 months). Selection of mating pairs was random from a pool of 48 males and 48 females allocated for a given day of the month. *bud13*^Δ7/Δ7^, *rbmx2*^Δ16/Δ16^, *snip1*^Δ11/Δ11^ were obtained through natural mating of heterozygous *bud13*^*+/* Δ7^, *rbmx2*^*+/* Δ16^, *snip1*^*+/* Δ11^ mutants, respectively (see below gene editing using CRISPR-Cas9). Tg(*dlx6a-1*.*4kbdlx5a/dlx6a*:*GFP*) lines [45,46] were obtained from the laboratory of Marc Ekker and Tg(*vglut*:*DsRed*) [47] from the laboratory of Joseph Fetcho.

Embryos were analyzed using a Zeiss Axioimager M1 and Discovery microscopes and photographed with a Zeiss Axiocam digital camera. Images were processed with Zeiss AxioVision 3.0.6.

### Confocal microscopy imaging and data processing

Whole-mount embryos were imaged in vivo by confocal microscopy (Leica TCS SP8 systems, Yale Center for Cellular and Molecular Imaging.) Mutant embryos and their wild-type siblings were scored at 30–32 hours post fertilization stage. Embryos were anesthetized using Tricaine and mounted in 0.6% agarose at an orientation where the frontal view of the brain was imaged. Embryos were imaged at 40x (1.3 Oil) using Z-stacks ranging from 70.41–96.02 μm, with a z stepping size of 0.4 μm. Z-stacks started at the first appearance of the GABAergic cells (GFP-labeled) and ended where GABAergic cells (GFP-labeled) could no longer be visualized. Each xy plane spanned 227.94 μm with a pixel size of 0.075 μm. Maximum intensity projections were shown for all confocal images. Images were processed using Fiji [48], Imaris (Bitplane) and Huygens deconvolution software (Scientific Volume Imaging). Figures were assembled using Illustrator (CC, Adobe). To quantify the number of glutamatergic (labeled in DsRed) and GABAergic cells (labeled in GFP) in the *bud13*^Δ7/Δ7^ mutant and their wild-type siblings respectively, two blinded raters segmented the raw z-stack images using ImarisCell module (Bitplane) and computationally counted the segmented cells in each channel (GFP and DsRed). Identical thresholds and parameters were applied to all samples for segmentation processing. Since the quantification performed by both independent raters yield consistent fold change in the respective cell counts between the *bud13*^Δ7/Δ7^ mutant and their wild-type siblings. Only one set of the analyzed results was displayed. Statistical analyses were conducted using Prism 6 (Graphpad).

### Acridine orange staining

To visualize apoptotic cells, vital dye acridine orange (Sigma) was used in live and dechorionated embryos. Embryos were incubated 2 minutes in PBS pH 7.1 with 2 ug/ml of acridine orange in the dark. After 3 brief washes in PBS, the embryos were placed in plates with 1% agarose and viewed with fluorescence microscopy, using the FITC filter set 1 [49].

### Plasmids constructs

Zebrafish *bud13*, *rbmx2* and *snip1* ORFs were PCR amplified (S4 Table) using cDNA from 2 and 6 hpf zebrafish embryos and cloned in pSP64T (*bud13*) in pT3TS [50] (*rbmx2* and *snip1*). *bud13* PCR product was cut using *NotI* and *EcoRI* and ligated into pSP64T. *rbmx2* and *snip1 PCR* products were cut with *NcoI* and *SacII* and ligated into pT3TS. To optimize Kozak sequence, the forward oligonucleotide used for *rbmx2* ORF introduced extra aminoacid in the 2nd position (GGC). Human *bud13* ORF was cloned in pSCDest [51] using gateway gene cloning system (Thermo Fisher Scientific). Final constructs were confirmed by sequencing. To generate mRNAs, the template DNA was linearized using *XbaI* (pT3TS), *BamHI* (pSP64T) or *KpnI* (pSCDest) and capped mRNA was synthetized using the mMessage mMachine T3 (pT3TS), or SP6 (pSP64T and pSCDest) kit (Ambion), respectively and in accordance with the manufacturer’s instructions. *In vitro* transcribed mRNAs were DNAse treated and purified using the RNeasy Mini Kit (Qiagen). All mRNA rescued the mutant phenotypes when 50–100 pg were injected in one cell stage embryo.

The plasmid (modified from [52]) for intron retention validation *in vivo*, pTol2(hsp-MCS-polyA, CMV-eGFP-SV40), is a Tol2 transposon-based, bipartite construct consisting of heat-shock promoter (hsp), a multiple cloning site (MCS), to insert the desired validation cassette, flanked by *Xenopus* globin UTR and polyadenylation tail (polyA) as well as a cis-linked CMV promoter and SV40 poly(A)-regulated eGFP reporter. Briefly the plasmid was built as following: pT2(kop:Cre-UTRnos3,CMV:eGFP) [52] was cut with Kpn1 to remove kop:Cre-UTRnos3. A synthetic DNA fragment containing HSP, 5’UTR *Xenopus* globin, MCS and 3’UTR *Xenopus* globin was obtained from Integrated DNA Technologies (IDT) and PCR amplified with specific primers (S4 Table). In-Fusion cloning protocol (Clontech) was performed using the cut vector and the PCR product to get the backbone vector pTol2(hsp-MCS-polyA, CMV-eGFP-SV40). Cassette for validation were obtained as synthetic DNA fragments (Integrated DNA Technologies, IDT) or amplified from genomic DNA (see S4 Table for details) and cloned directionally in frame with XhoI and SacII. Final constructs were confirmed by sequencing.

### Morpholino, plasmid injections and gene editing using CRISPR-Cas9

A morpholino targeting *bud13* mRNA start codon was obtained from Gene Tools and re-suspended in nuclease-free water. 1 nl of morpholino solution (0.6mM) was injected into wild-type dechorionated embryos at the one-cell stage.

A mix of 4 plasmid (15 pg/embryo) with the desired cassette (minigene), 2 predicted as no retained (*serpinb1l3*, *col1a2*) and 2 predicted as retained (*wdr26b*, *ptch2*) upon RES loss-of-function was injected in 1 cell stage embryo together with Tol2 mRNA (Addgene plasmid #31831) (33 pg/embryo). Embryos were sorted in *bud13*^*∆7/∆7*^ and *bud13*^*+/*?^ at the onset of the phenotype (~ 28–30 hpf). Minigene expression under HSP promoter was induced by heat shock during 4 hs and then GFP positive embryos were collected for RT-PCR. PCR was performed using the specific Fw primer an a universal Rv globin (S4 Table, Fig 6C and 6D and S12 Fig).

CRISPR-Cas9-mediated gene editing was performed as described previously [53]. Briefly, 3 different sgRNAs (20 pg each) targeting *bud13* gene (S4 Table) were co-injected together with 100 pg of mRNA coding for zebrafish codon optimized Cas9-nanos in one-cell stage embryos (S1A Fig). Cas9-nanos concentrates gene editing in germ cells and increases the viability of injected embryos [20]. F_0_ founders were mosaic and they were backcrossed with wild-type fish and then F1 fish were genotyped using their corresponding oligos per target site (S4 Table). Heterozygous adult fish *bud13*^*+/* Δ7^ (Fig 1) were selected to generate *bud13*^Δ7/Δ7^ mutants. Similar approach was followed to generate *rbmx2* and *snip1* mutants but injecting 2 sgRNAs (S4 Table and Fig 1).

### *In situ* hybridization

*krox20*, *shh* and *pax2a* in situ probes were previously described [54–56]. RES antisense and sense digoxigenin (DIG) RNA probes were generated by in vitro transcription in 20 μl reactions consisting of 100 ng purified PCR product (8 μl), 2 μl DIG RNA labelling mix (Roche), 2 μl ×10 transcription buffer (Roche), and 2 μl T7/T3 (antisense probes) and SP6 (sense probes) RNA polymerase (Roche) in RNase-free water and purified using a Qiagen RNEasy kit. In situ protocol was followed as detailed previously [57]. To reduce variability, wild-type sibling and *bud13*^Δ7/Δ7^ embryos were combined in the same tube during in situ hybridization and recognized based on their phenotype. Before photo documentation, embryos were cleared using a 2:1 benzyl benzoate:benzyl alcohol solution. Images were obtained using a Zeiss stereo Discovery V12.

### RNAseq library, reverse transcription PCR (RT–PCR) and qPCR

Total RNA from 32 hpf *bud13* ^*Δ7/Δ7*^, 48 hpf *rbmx2* ^*Δ16/Δ16*^, 48 hpf *snip1* ^*Δ11/Δ11*^ embryos and their corresponding siblings was extracted using Trizol (ten embryos per condition). Strand-specific TruSeq Illumina RNA sequencing libraries were constructed by the Yale Center for Genome Analysis. Samples were multiplexed and sequenced on Illumina HiSeq 2000/2500 machines to produce 76-nt paired-end reads.

RNA used for intron retention validation experiments was treated with TURBO DNase (Ambion) for 30 min at 37°C and extracted using phenol chloroform. Then, Polyadenylated RNAs were purified using Oligo d(T)25 Magnetic Beads (Invitrogen) following manufacter recommendations. cDNA was generated by reverse transcription with random hexamers using SuperscriptIII (Invitrogen). RT–PCR reactions were carried out at an annealing temperature of 59°C for 35–40 cycles. PCR products were run in a 1.5% agarose gel. Primers are listed in the S4 Table.

For the qPCR experiment, total RNA was extracted as described above. GFP and dsRED mRNAs were used as spike-in RNA controls and 1 μg of total RNA was used to generate cDNA. 5 μl from a 1/50 dilution of the cDNA reaction was used to determine the levels of p53 in a 20 μl reaction containing 1 μl of each oligo forward and reverse (10 μM) (S4 Table), using Power SYBR Green PCR Master Mix Kit (Applied Biosystems) and a ViiA 7 instrument (Applied Biosystems). PCR cycling profile consisted of incubation at 50°C for 2 min, followed by a denaturing step at 95°C for 10 min and 40 cycles at 95°C for 15 s and 60°C for 1 min. Primers are listed in S4 Table.

### Genotyping

Zebrafish embryos or a small amount of tissue from the end of the tail were used to extract DNA [58]. Briefly, embryos or fin clipped were incubated in 80 μl of NaOH 100mM at 95°C for 15 min producing a crude DNA extract, which was neutralized by the addition of 40 μl of 1 M Tris-HCl, pH 7.4 (Sigma-Aldrich). 1 μl of this DNA extraction was used as a template for PCR reactions using the primers described in S4 Table.

### Gene expression analyses

Gene expression levels for each condition were calculated from RNA-seq data using the cRPKM metric (corrected-for-mappability Reads Per Kilobasepair of uniquely mappable positions per Million mapped reads [59]. For this, a reference transcript per gene was selected from the Ensembl version 80 annotation for *Danio rerio* using BioMart (25,935 genes in total, S5 Table) and uniquely mappable positions for each transcript were calculated as previously described [59]. Quantile normalization of cRPKM values was done with ‘normalizeBetweenArrays’ within the ‘limma’ package.

To identify differentially expressed genes, we first filtered out genes that did not have cRPKM > 2 in all sibling control or all mutant samples and genes whose quantification was not supported by at least 50 read counts in at least 1 sample. Next, differentially expressed genes were defined as those that showed a fold change in expression of at least 1.5 in all 3 mutants and a fold change of at least 2 in 2 out of the 3 control vs. mutant individual comparisons (*bud13*, *rbmx2* and *snip1*). Gene Ontology analysis was performed with the online tool DAVID (https://david.ncifcrf.gov/ Version 6.8) using as background all genes that passed the initial filters (minimum expression and read count).

### Genome-wide analysis of intron retention

Annotated introns for each reference zebrafish transcript in Ensembl version 80 were extracted and those that overlapped with other genes were removed yielding a total of 182,017 valid introns. To calculate the percent of intron retention (PIR) for each intron in a given RNA-seq sample, we used our previously described pipeline [22] with the following modification: to calculate intron removal, all exon-exon junctions supporting the splicing of the intron were used and not only those formed between the two neighboring exons. This was done to avoid false positives in the case of introns associated to cassette exons or other alternative splicing events. For all analysis, only introns with sufficient read coverage across the six samples were considered (at least 15 reads supporting the inclusion of one splice site and 10 of the other, or a total of 15 reads supporting splicing of the intron).

To define the confident set of highly affected introns, potential false positives were filter out by comparing the density of the mapped reads in the introns bodies in the mutant vs the control. For this purpose, we extracted all intronic sequences and calculated the number of uniquely mappable positions per intron following a similar strategy to that used to calculate cRPKMs [59] (see above). Specifically, every 50-nucleotide (nt) segment in 1-nucleotide sliding intronic windows was mapped to a library of full-length intronic sequences plus the whole genome, using bowtie with–m 2 –v 2 parameters (every intronic segment must map at least twice, to its own individual intron sequence and to the corresponding position in the whole genome). Segments that mapped more than twice were considered as multi-mappable positions, whereas those that did not map (e.g. due to undetermined (N) nucleotides in the assembly) were considered as non-mappable. The number of uniquely mappable positions of an intron is defined as the total number of segments minus multi- and non-mappable positions. Next, each RNA-seq sample was mapped to the same library of intronic plus full genomic sequences using–m 2 –v 2 to obtain the unique intronic reads counts. However, to minimize potential artifacts derived from the heterogeneity of the intronic sequences (e.g. high number of reads mapping to a transposable element or an expressed nested gene), if a given intronic position showed a read count more than five times higher than the median read count of the whole intron, then the read count of this position was set to 5 × median (if the median read count was 0, then the maximum read count for any given position was set to 5). Finally, ciRPKM scores were calculated (corrected-for-mappability intronic Reads Per Kilobasepair of uniquely mappable positions per Million mapped reads) for each intron and condition by dividing this number of counts by the number of uniquely mappable positions in that intron.

With this information, the set of confidently retained introns upon RES complex disruption (“RESdep” introns) were defined as those introns with ∆PIR > 15 and at least a 1.5-fold net increase in read density in the intron body calculated as:

$$\left[ciRPKM_{mut}/\left(100‑PIR_{mut}\right)\right]/\left[ciRPKM_{sib}/\left(100‑PIR_{sib}\right)\right]$$

in at least 2 out of 3 mutants (1,413 introns in total). As a control, we also define a set of confidently non-retained introns as those with a ∆PIR < 0.5 in the 3 mutants (“Ctr” set; 5,577 introns).

To analyze the number of retained introns in the 3 mutants and the inter-mutant overlaps Euler APE-3.0.0 software [60] was utilized.

### Analysis of intron-associated features

To investigate the impact of non-sense mediated decay (NMD) on global intron retention upon RES mutation, all introns were separated as last or non-last introns (of the reference transcript) and between those predicted to trigger and not to trigger NMD. An intron was predicted to trigger NMD if its retention generated an in-frame stop codon that is located further than 50 nts upstream of an exon-exon junction [61]. By definition, last introns cannot trigger NMD. ∆PIR values were plotted as boxplots for each category, and two-sided Wilcoxon Sum Rank tests were used to evaluate statistical differences between the distributions.

To identify features discriminating introns highly retained upon RES depletion from un-retained/un-affected introns, we compared the sets of confidently introns (“RESdep” in Fig 5.) with control introns (“Ctr” in Fig 5.). Moreover, as introns that are predicted not to trigger NMD are expected to be more often accumulated unspecifically, two subsets for “RESdep” introns were generated: (i) those introns in genes with more than five introns, are not the last three introns of the gene, and that are predicted to trigger NMD (“NMD”, 577 introns); and (ii) predicted not to trigger NMD or cause a frame shift upon inclusion, unless they are the last intron of the gene (“no-NMD”, 569 introns). For these different sets of introns, 44 features were extracted (S3 Table), including intron and exon length and GC content, strength of 3' and 5' splice sites, branch point (BP) related features, and transcript length, using custom scripts in combination with the following two external tools: MaxEntScan scripts for determining the strength of 3' and 5' splice sites [62]; and SVM-BPfinder software for determining BP related features (BP strength, distance from BP to 3' splice site, and pyrimidine track length) [26]. For the latter analysis, the 150 nts upstream of the 3' splice sites were extracted and these sequences were used as input for SVM-BPfinder. Furthermore, we recorded the highest log-score of the SF1 position weight matrix binding model across these 150-nt intronic sequences [25].

### Logistic regression model and evaluation of feature discrimination capacity

We applied logistic regression models to the discrimination between differentially retained introns (retained) and non-differentially retained introns (control) upon RES mutations. We focused on the set of confidently retained introns (“RESdep”, 1,409 introns) versus control introns with an absolute ∆PIR < 0.5 in the three mutants (“Ctr”, 5,565; we removed 9 introns for which we could not determine all features). The binary response variable of the logistic regression models indicates for each intron if it belongs to the retrained or control group. As predictors we used 30 quantitative and qualitative features (S3 Table), including intronic and exonic characteristics, position along the transcript and gene expression in wild type conditions, among others. The binomial logistic regression models were learned using Lasso variable selection [63–65] available in R through library glmnet (2.0.10), and the generalized linear model function glm from the R stats library.

To investigate the overall classification performance, we randomly partitioned the data set “RESdep” of retained introns into 90%/10% (i.e., 1268/141) training/test data, and randomly sampled the same amount of training/test data from the control “Ctr” data set. We used the training data to learn a logistic regression model with Lasso variable selection and tested it on the test data. Next, to evaluate how well this model classifies specific subsets of "RESdep" introns and retained introns specific for each mutation, we applied the model trained with “RESdep” vs “Ctr” data having held fixed its parameters to the classification of the 141 control test-introns vs. 141 retained introns subsampled from the following sets: (i) “RESdep_∆PIR10” introns from the “RESdep” set with ∆PIR > 10 in all three mutants (871 introns); (ii) “NMD”, introns from the “RESdep” set predicted to trigger NMD when retained (574 introns); (iii) “bud13”, introns with ∆PIR>15 upon bud13 mutation at 32 hpf (2,363 introns); (iv) “rbmx2”, introns with ∆PIR>15 upon rbmx2 mutation at 48 hpf (2,186 introns); and (v) “snip1”, introns with ∆PIR>15 upon snip1 mutation at 48 hpf (2,675 introns). We repeated this procedure, including model training and classification of test data, 10,000 times and report average ROC curves (Fig 6A) and average model coefficients for each feature extracted from the trained models. These averages indicate the direction of the effect (e.g. positively [blue] or negatively [red] associated with retention upon RES mutation; Fig 6B; S10B Fig and S3 Table).

To study the potential of each feature to contribute to the discrimination between the "RESdep" and "Ctr" intron sets, we randomly partitioned the dataset of retained introns into 90%/10% (i.e., 1268/141) training/test data, and randomly sampled the same amount of training/test data from Ctr. Using the training data, we learned logistic regression models without Lasso variable selection using only a single feature at a time neglecting all other features. The test data were used to determine the AUC. This experiment was repeated 10,000 times and we report average AUCs for each feature in Fig 6B and S10B Fig. In addition, the fraction of the null deviance that was reduced by each single-feature model was recorded, and the average reductions of the null deviance for each feature are reported in S3 Table.

## Supporting information

S1 Table (XLSX)

S2 Table (XLSX)

S3 Table (XLSX)

S4 Table (XLSX)

S5 Table (TXT)

S6 Table (TAB)

S1 Summary Statistics (XLSX)

S1 Fig RES complex is required during zebrafish development. **(A)** Scheme illustrating the Cas9-nanos 3′-UTR strategy [20]. The nanos' 3′-UTR concentrates the expression of Cas9 in the germ cells (green circles). **(B-C)** Bright field microscopy of RES mutant embryos and their corresponding phenotypically wild type sibling (WT), treated with PTU to avoid melanocyte production, in lateral view **(C)** or magnification (B, for *rbmx2* and *snip1*). Increased levels of apoptosis, predominantly in the head, are observed upon RES loss-of-function. (scale bar: 0.5mm at 48 hpf; 0.35mm at 32 hpf). **(D)** UCSC genome tracks showing mRNA levels of RES complex members at 2 cell and 48 hpf stages. **(E)** RT-qPCR showing *p53* mRNA levels. Error bars represent SD of the mean from two independent biological replicates (n = 10 embryos per biological replicate). A *p53* up-regulation in the mutants compared to phenotypically WT siblings correlates with the increased cell dead observed in the brain. **(F)** In situ hybridization showing RES components sense (top) and antisense (bottom) probes. Lack of expression in the sense probes show the specifity of the RES expression pattern in the zebrafish developing embryos.
(TIF)

S2 Fig bud13 knock-down show stronger cell death phenotype. **(A)** Lateral view of WT embryos injected with 0.6mM of morpholino antisense oligonucleotide against *bud13* mRNA (MO*bud13*) showing different levels of developmental defects (types I to III). Phenotypes are fully rescue with human *hBUD13* mRNA. (scale bar: 0.5mm). WT: represent phenotypically wild type sibling from the same mutant fish line. Stronger phenotype is likely due to a depletion of the maternal contribution. **(B)** *bud13* mutant embryos fully rescued by providing 75 pg of *hBUD13* mRNA, suggesting that Bud13 function may be conserved across vertebrates (scale bar: 0.5mm). WT: represent phenotypically wild type sibling from the same mutant fish line. **(C)** 48 hpf, *bud13^Δ7/Δ7^* embryos showing a similar phenotype to *rbmx2^Δ16/Δ16^* and *snip1^Δ11/Δ11^* when partly rescued by injection of 2.5 pg of *hBUD13* mRNA (scale bar: 1mm). WT: represent phenotypically wild type sibling from the same mutant fish line.
(TIF)

S3 Fig CNS molecular markers show mild differences in *bud13^Δ7/Δ7^*. *In situ* hybridization showing expression pattern of *shh* (notochord and floor plate), *krox20* (*egr2a*; rhombomere 3 and 5) and *pax2a* (anterior midbrain-hindbrain boundary and hindbrain neurons) in WT (top) and mutant (bottom) embryos.
(TIF)

S4 Fig Transcript expression levels and Gene ontology analysis. **(A)** Biplot comparing transcript expression levels in RES mutants and their corresponding phenotypically WT siblings. Genes up- or down-regulation were defined as having a fold change in expression of at least 1.5 in all three mutants and at least 2 for two out the three control vs. mutant individual comparisons (*bud13*, *rbmx2* and *snip1*) (log10 cRPKM). **(B-C)** DAVID cluster analysis of enriched GO annotations for down-regulated (B) or up-regulated genes (C) in RES mutants compared with wild-type siblings. **(D)** Barplots showing expression values (using the cRPKM metric) for up- regulated genes associated with cell death (*p53*, *caspase8* and *puma*). The significant up-regulation in the three mutants correlates with the increased cell dead observed in the developing brain.
(TIF)

S5 Fig Differentially retained introns detected by RT-PCR. Sequencing read density across the *ptch2* **(A)** and wdr26b **(B)** loci (upper panels). RNA- seq signal increases strongly (∆PIR>15) in RES mutants only on specific introns (dark blue). RT-PCR assays validating the increased retention (dotted square box) in *bud13*, *rbmx2* and *snip1* mutants compared with the corresponding phenotypically WT siblings (lower panel).
(TIF)

S6 Fig Gene ontology and gene expression analysis of retained introns. **(A)** DAVID cluster analysis of enriched GO annotations for genes that contain introns with highly increased retention (∆PIR>15) in at least two of the RES mutants. **(B)** Boxplots showing fold change in expression (FC-expr) for genes containing increased retention (∆PIR>15) compared with those genes in which all transcripts are not affected (∆PIR<2) in the RES mutants. *P*-values were calculated using Wilcoxon rank-sum tests. Genes with at least one strongly retained intron (ΔPIR>15) had significantly decreased expression in the mutants compared to genes with no substantial change in intron retention (ΔPIR<2). **(C)** Boxplots illustrating differences in intron retention between genes that were up-regulated (Up), down-regulated (Down) or did not show significant expression changes (Unaffected). Number of introns in each category: Up = 1,603; Down = 1,623; Unaffected = 69,646. *P*-values were calculated using Wilcoxon rank-sum test. Genes that were differentially expressed in the three mutants (down- or up-regulated) show significantly higher retention (higher ΔPIR) in the mutants compared to their WT-looking siblings. **(D)** Overlap between differentially expressed genes (up and down-regulated) and genes harboring retained introns. *P*-values were calculated using Wilcoxon rank-sum tests: down vs IR *P* = 0.4033681; up vs IR *P* = 0.000117.
(TIF)

S7 Fig Intron enrichment. Stacked bar plot showing enrichment for last introns **(A)** and introns predicted not to trigger NMD upon inclusion **(B)** among the RES-dependent (RES-dep) introns. On the contrary, introns that are not affected by RES depletion (Ctr) are depleted for these types of introns. P-values were calculated using Fisher exact test. **(C)** Stacked barplots showing the percentage of introns affected by *bud13*, *rbmx2* or *snip1* mutation using different ∆PIR cutoffs. **(D)** Euler diagram showing the overlap of skkiped exons (∆PIR>5 and ∆PIR>15) affected by *bud13*, *rbmx2*, and *snip1* mutants.
(TIF)

S8 Fig Extended features of the RES-dependent introns. **(A)** Boxplots showing the distribution of the median nucleotide (nt) distance from the top 3 predicted branch points (BP) to the 3’ splice site for each intron category. **(B)** Boxplots of the highest score for the human SF1 position weight matrix (PWM) in the 3’ intronic region (see Methods for details). **(C-D)** Boxplots showing the GC content ratio between up-stream (C) or down-stream (D) exons vs the retained introns. The lower ratio of no- NMD introns, which are enriched in last introns, is caused by the generally low GC content of last exons overlapping the 3' UTR. (***P ≤ 0.001, Mann-Whitney-U test).
(TIF)

S9 Fig Intron length and GC content genome wide analysis. **(A)** Boxplots showing the degree of change in intron retention (∆PIR) according to intron length. Number of introns per nucleotide bin: ≤100 = 13,059; 101–300 = 13,332; 301–1,000 = 11,808; 1,001–2500 = 18,164; >2500 = 16,563. **(B)** Boxplots illustrating the degree of change in intron retention (∆PIR) according to intron GC content. Number of introns per bin: 0–0.3 = 14663; 0.3–0.35 = 27361; 0.35–0.4 = 21873; 0.4–0.45 = 6721; 0.45–1 = 2308. *P*-values were calculated using Wilcoxon rank-sum test.
(TIF)

S10 Fig Contribution of each feature to reduction of null deviance. **(A)** Logistic regression models were learned to discriminate between RESdep and Ctr introns with each feature individually and the fraction of the null deviance that was reduced was recorded. Values were averaged over 10,000 repeated holdout experiments. Training data sets consisted of 1,268 RESdep and 1,268 Ctr introns. Error bars indicate 95% confidence interval of reported averages. **(B)** Features call used in Fig 6B and in S10A Fig.
(TIF)

S11 Fig Validation of logistic regression model. **(A)** ∆PIR values for the top 100 introns based on their likelihood to be bud13-dependent and -independent, as predicted by the regression model. Only introns with no read coverage across the six RNA-seq samples were used for this analysis (108,470 introns). False positive rate (FPR) values are defined for retained introns to be the fraction of introns with ∆PIR<2, and for unaffected introns to be those with ∆PIR>5. **(B)** RT-PCR assays validating the predicted RES dependent and RES independent introns in *bud13*, *rbmx2* and *snip1* mutants compared with the corresponding phenotypically WT siblings (lower panel). Arrows indicate the retained intron. In the case of *arf6a* we could detect intron retention by RT-PCR only in the rbmx2 mutant. Unspecific upper band in *pde4ba*, *rpp38*, *znf644b* and *c8h17orf59* are likely DNA heteroduplexes. The numbers underneath the introns in the cartoons represent their lengths in bp.
(TIF)

S12 Fig RES-dependent and independent introns *in vivo* validation. **(A)** Intron predicted as RES independent were spliced in *bud13∆7/∆7* (*col1a2* and *serpinb1l3*) while RES dependent intron *wdr26b*, but not *ptch2*, was retained as predicted for the logistic regression model. **(B)** Scheme of the vector used in the validation assay. PCR product and primers used are depicted as specific Fw primer (blue arrow) and universal Rv primer (magenta arrow). All experiments were done using two independent biological replicates (rep#1 and rep#2).
(TIF)
