# Supplementary figures and images for: RES complex is associated with intron definition and required for zebrafish early embryogenesis
Source: PLoS Genet. 2018 Jul 3;14(7):e1007473. doi: 10.1371/journal.pgen.1007473 (PMC6047831; doi:10.1371/journal.pgen.1007473)

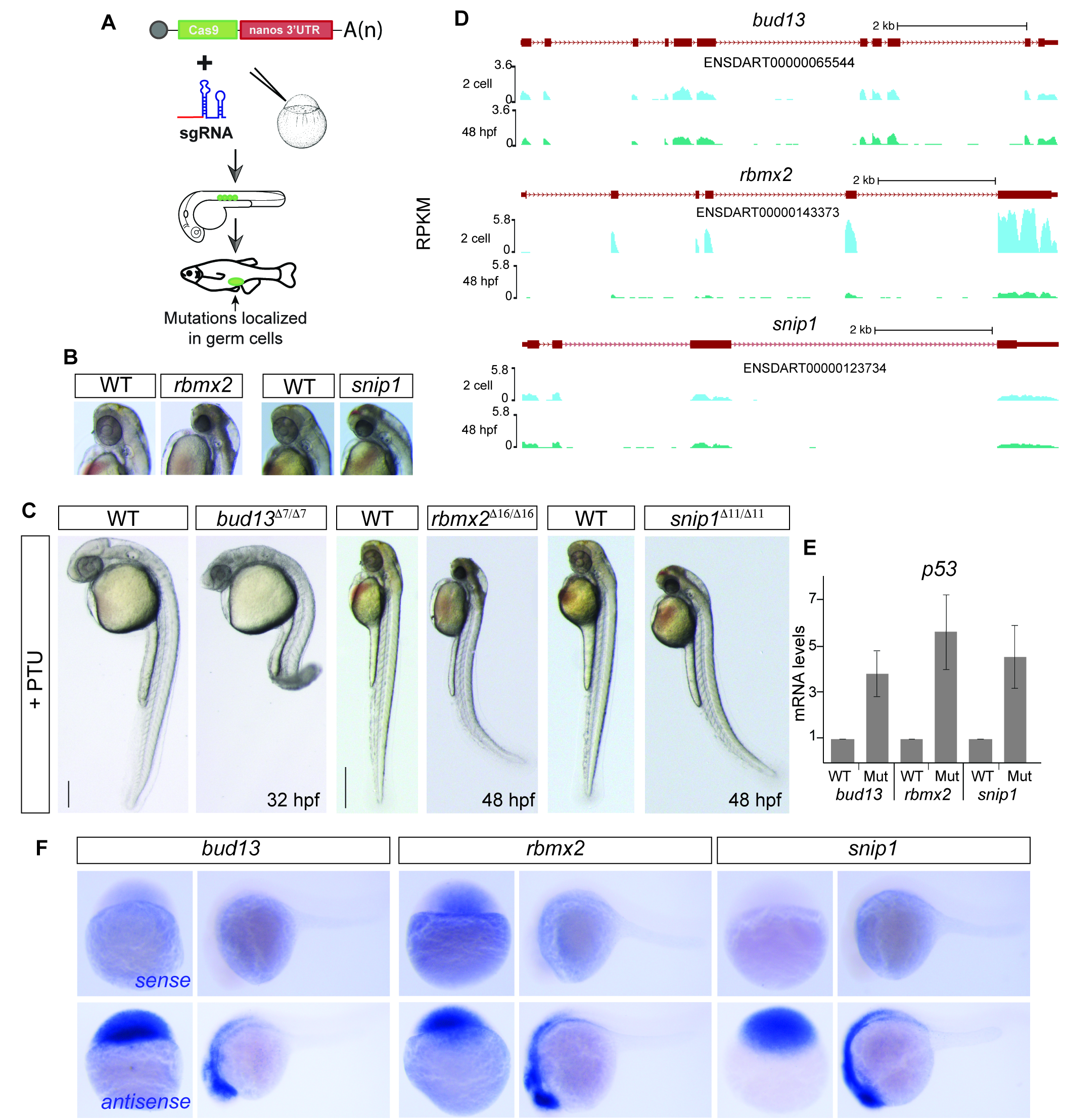

Supplement: S1 Fig — (A) Scheme illustrating the Cas9-nanos 3′-UTR strategy [20]. The nanos' 3′-UTR concentrates the expression of Cas9 in the germ cells (green circles). (B-C) Bright field microscopy of RES mutant embryos and their corresponding phenotypically wild type sibling (WT), treated with PTU to avoid melanocyte production, in lateral view (C) or magnification (B, for rbmx2 and snip1). Increased levels of apoptosis, predominantly in the head, are observed upon RES loss-of-function. (scale bar: 0.5mm at 48 hpf; 0.35mm at 32 hpf). (D) UCSC genome tracks showing mRNA levels of RES complex members at 2 cell and 48 hpf stages. (E) RT-qPCR showing p53 mRNA levels. Error bars represent SD of the mean from two independent biological replicates (n = 10 embryos per biological replicate). A p53 up-regulation in the mutants compared to phenotypically WT siblings correlates with the increased cell dead observed in the brain. (F) In situ hybridization showing RES components sense (top) and antisense (bottom) probes. Lack of expression in the sense probes show the specifity of the RES expression pattern in the zebrafish developing embryos. (TIF) [file pgen.1007473.s008.tif]

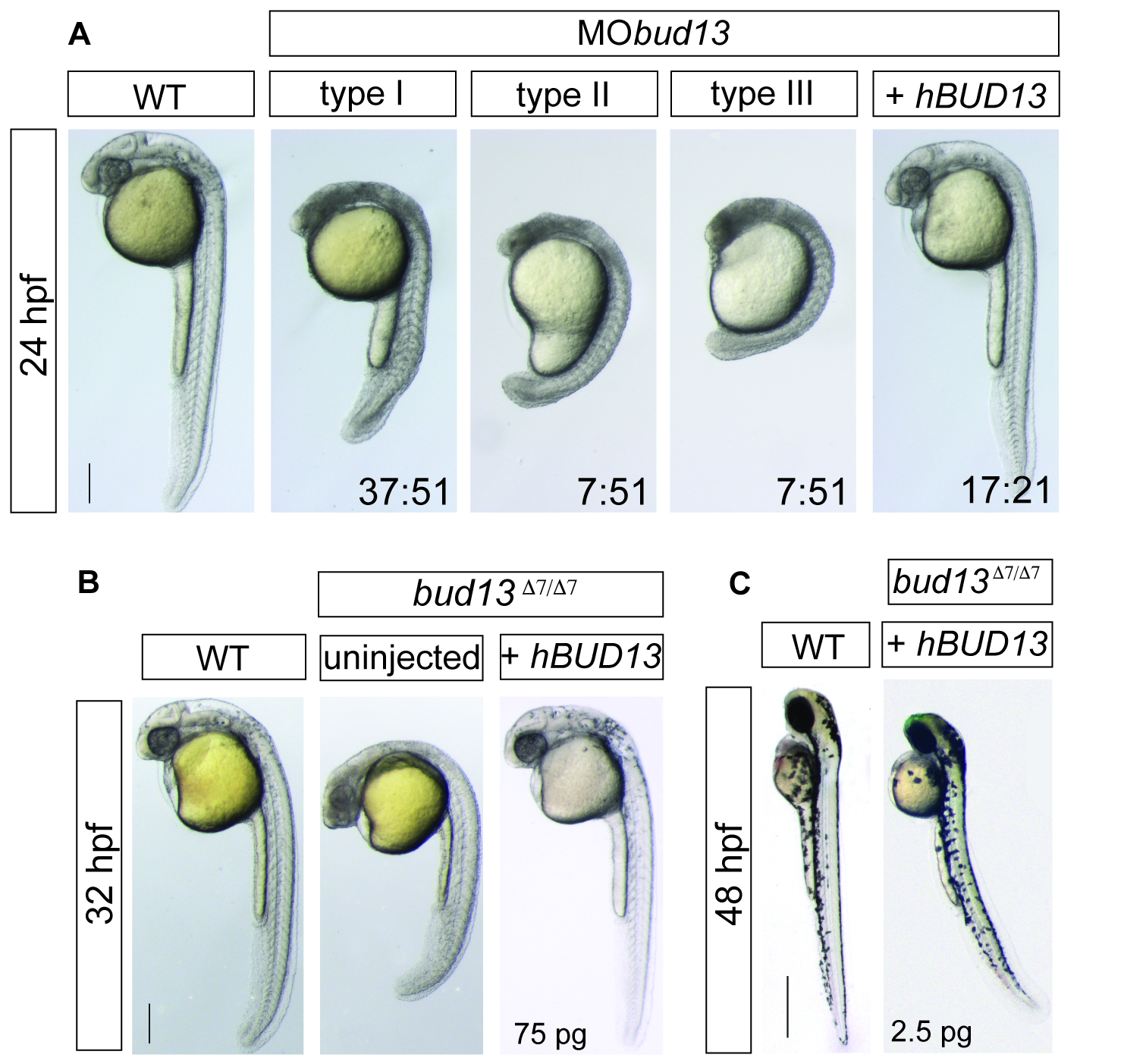

Supplement: S2 Fig — (A) Lateral view of WT embryos injected with 0.6mM of morpholino antisense oligonucleotide against bud13 mRNA (MObud13) showing different levels of developmental defects (types I to III). Phenotypes are fully rescue with human hBUD13 mRNA. (scale bar: 0.5mm). WT: represent phenotypically wild type sibling from the same mutant fish line. Stronger phenotype is likely due to a depletion of the maternal contribution. (B) bud13 mutant embryos fully rescued by providing 75 pg of hBUD13 mRNA, suggesting that Bud13 function may be conserved across vertebrates (scale bar: 0.5mm). WT: represent phenotypically wild type sibling from the same mutant fish line. (C) 48 hpf, bud13Δ7/Δ7 embryos showing a similar phenotype to rbmx2Δ16/Δ16 and snip1Δ11/Δ11 when partly rescued by injection of 2.5 pg of hBUD13 mRNA (scale bar: 1mm). WT: represent phenotypically wild type sibling from the same mutant fish line. (TIF) [file pgen.1007473.s009.tif]

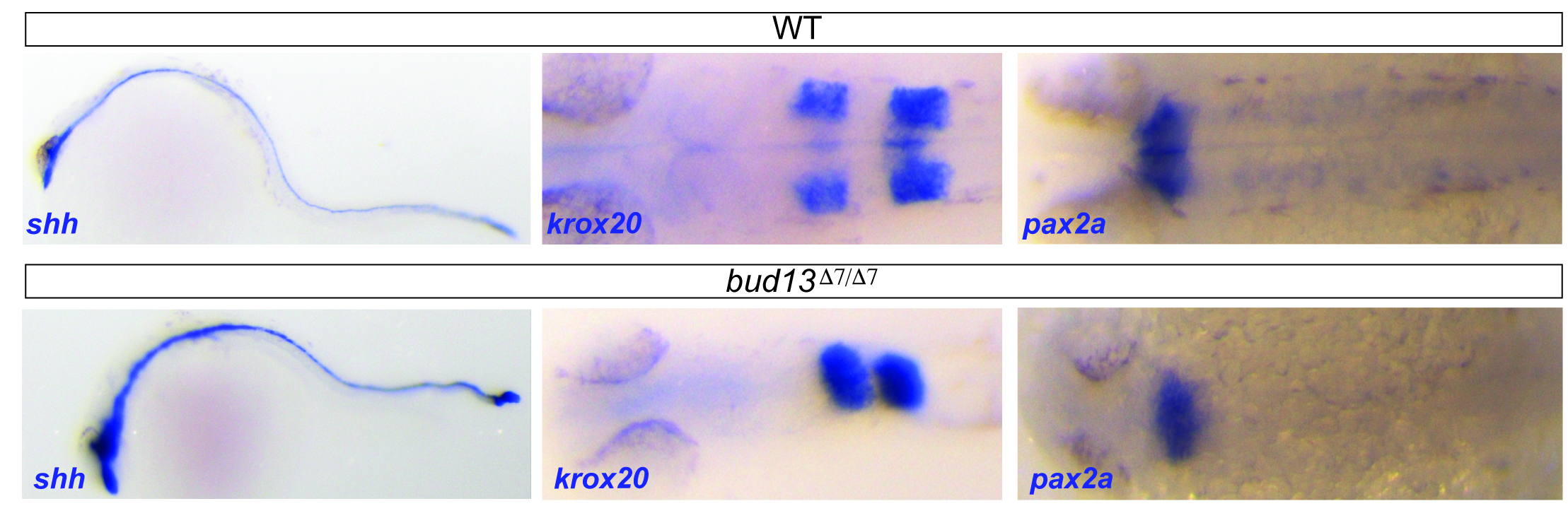

Supplement: S3 Fig — In situ hybridization showing expression pattern of shh (notochord and floor plate), krox20 (egr2a; rhombomere 3 and 5) and pax2a (anterior midbrain-hindbrain boundary and hindbrain neurons) in WT (top) and mutant (bottom) embryos. (TIF) [file pgen.1007473.s010.tif]

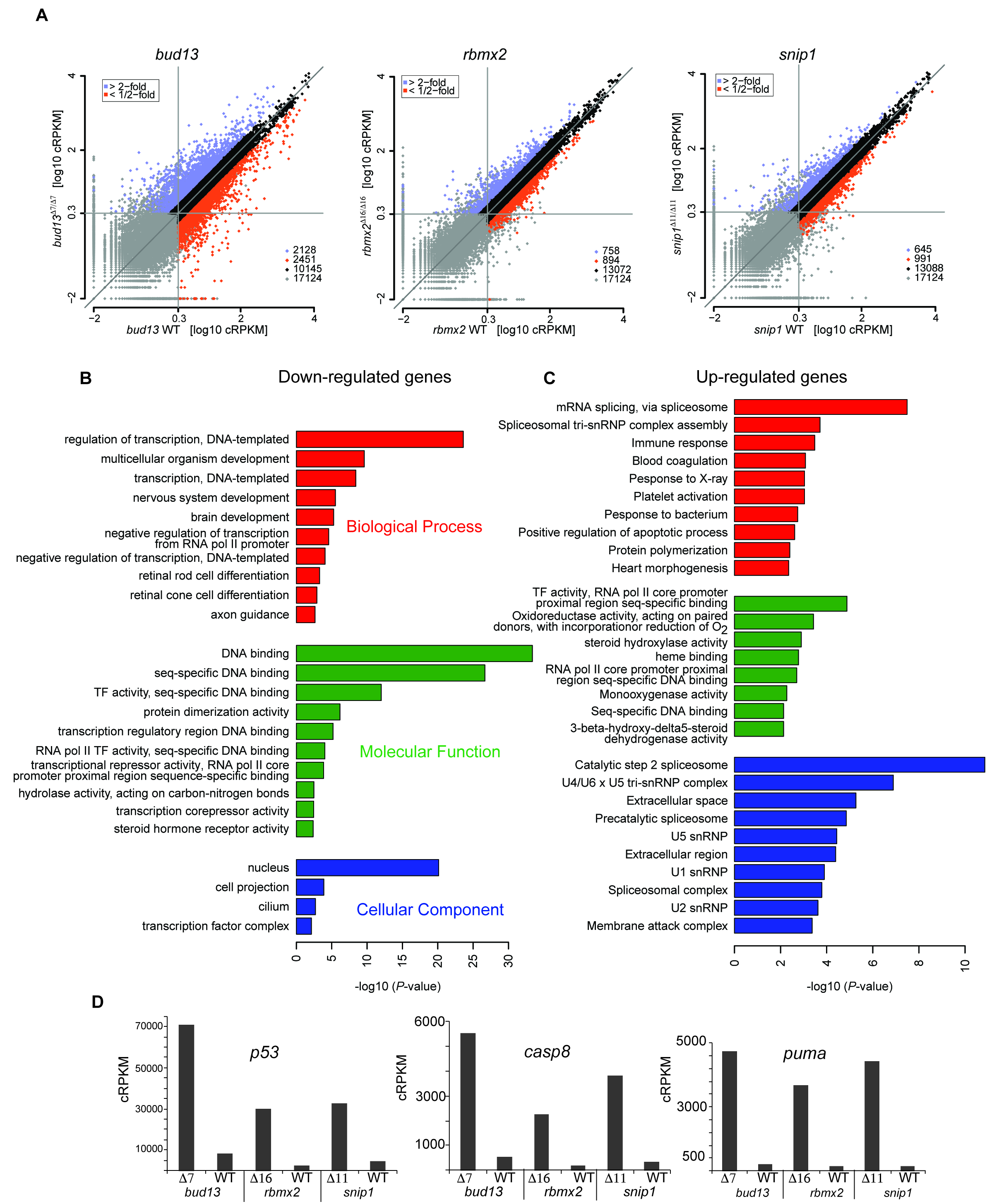

Supplement: S4 Fig — (A) Biplot comparing transcript expression levels in RES mutants and their corresponding phenotypically WT siblings. Genes up- or down-regulation were defined as having a fold change in expression of at least 1.5 in all three mutants and at least 2 for two out the three control vs. mutant individual comparisons (bud13, rbmx2 and snip1) (log10 cRPKM). (B-C) DAVID cluster analysis of enriched GO annotations for down-regulated (B) or up-regulated genes (C) in RES mutants compared with wild-type siblings. (D) Barplots showing expression values (using the cRPKM metric) for up- regulated genes associated with cell death (p53, caspase8 and puma). The significant up-regulation in the three mutants correlates with the increased cell dead observed in the developing brain. (TIF) [file pgen.1007473.s011.tif]

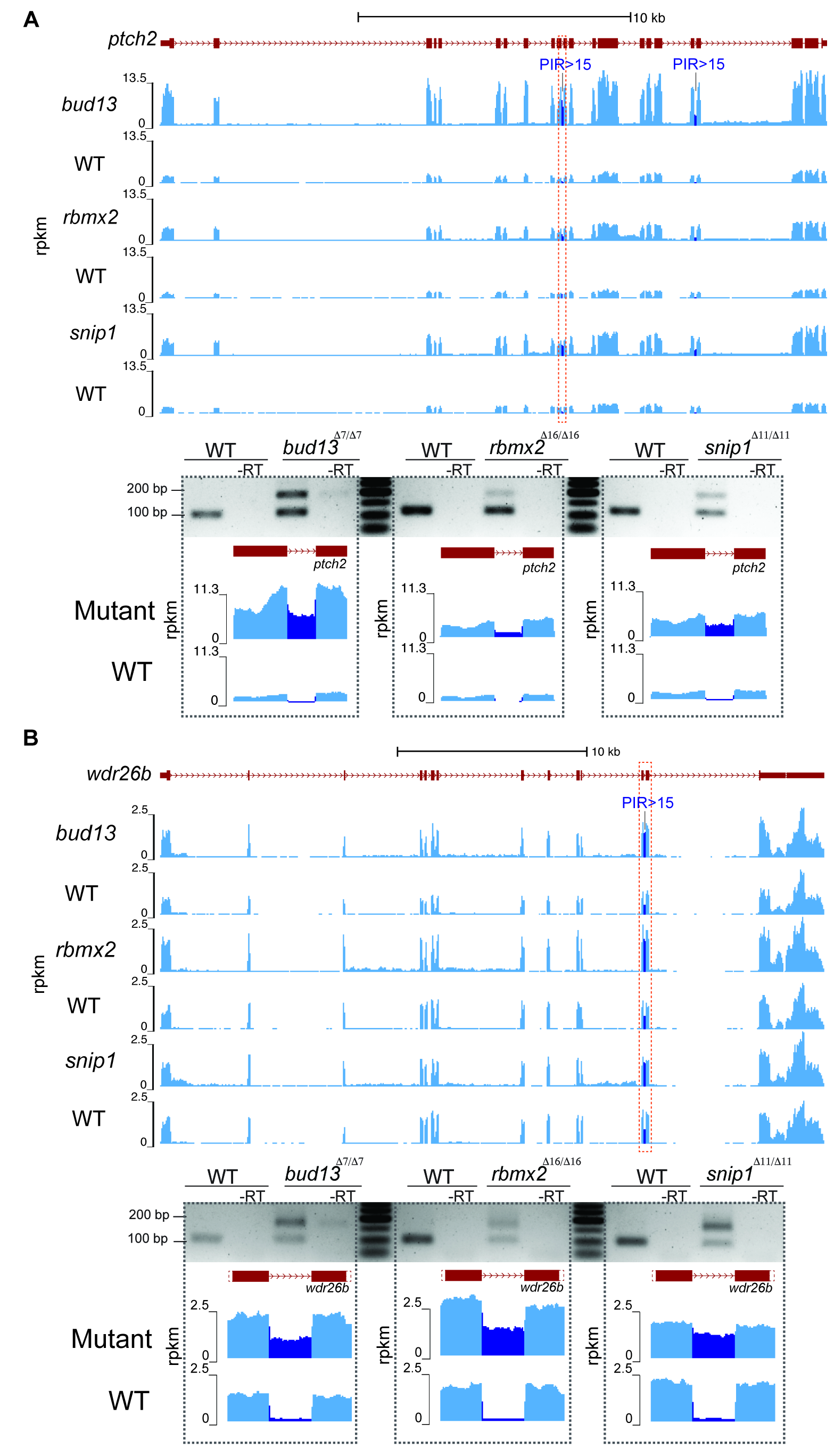

Supplement: S5 Fig — Sequencing read density across the ptch2 (A) and wdr26b (B) loci (upper panels). RNA- seq signal increases strongly (∆PIR>15) in RES mutants only on specific introns (dark blue). RT-PCR assays validating the increased retention (dotted square box) in bud13, rbmx2 and snip1 mutants compared with the corresponding phenotypically WT siblings (lower panel). (TIF) [file pgen.1007473.s012.tif]

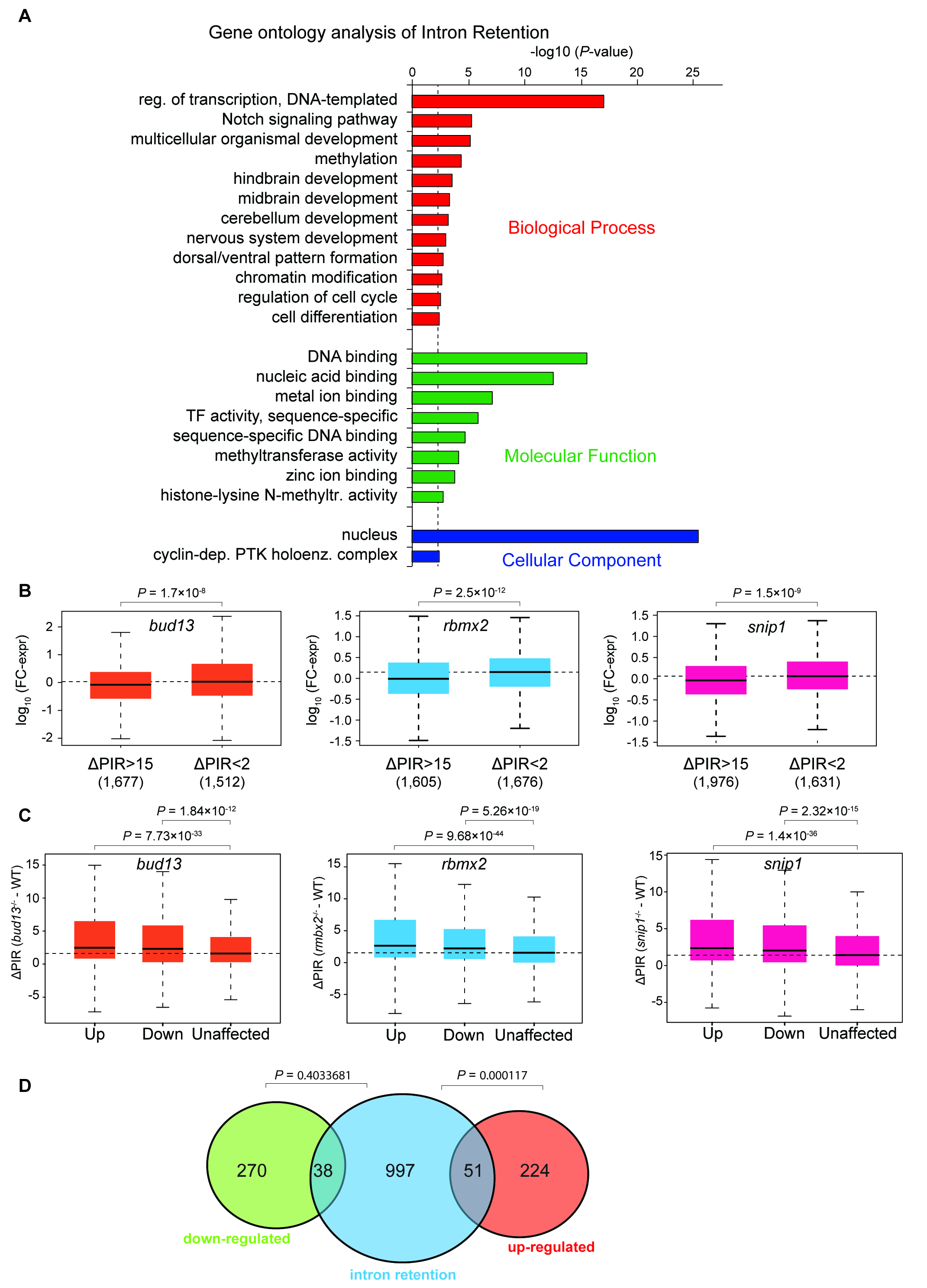

Supplement: S6 Fig — (A) DAVID cluster analysis of enriched GO annotations for genes that contain introns with highly increased retention (∆PIR>15) in at least two of the RES mutants. (B) Boxplots showing fold change in expression (FC-expr) for genes containing increased retention (∆PIR>15) compared with those genes in which all transcripts are not affected (∆PIR<2) in the RES mutants. P-values were calculated using Wilcoxon rank-sum tests. Genes with at least one strongly retained intron (ΔPIR>15) had significantly decreased expression in the mutants compared to genes with no substantial change in intron retention (ΔPIR<2). (C) Boxplots illustrating differences in intron retention between genes that were up-regulated (Up), down-regulated (Down) or did not show significant expression changes (Unaffected). Number of introns in each category: Up = 1,603; Down = 1,623; Unaffected = 69,646. P-values were calculated using Wilcoxon rank-sum test. Genes that were differentially expressed in the three mutants (down- or up-regulated) show significantly higher retention (higher ΔPIR) in the mutants compared to their WT-looking siblings. (D) Overlap between differentially expressed genes (up and down-regulated) and genes harboring retained introns. P-values were calculated using Wilcoxon rank-sum tests: down vs IR P = 0.4033681; up vs IR P = 0.000117. (TIF) [file pgen.1007473.s013.tif]

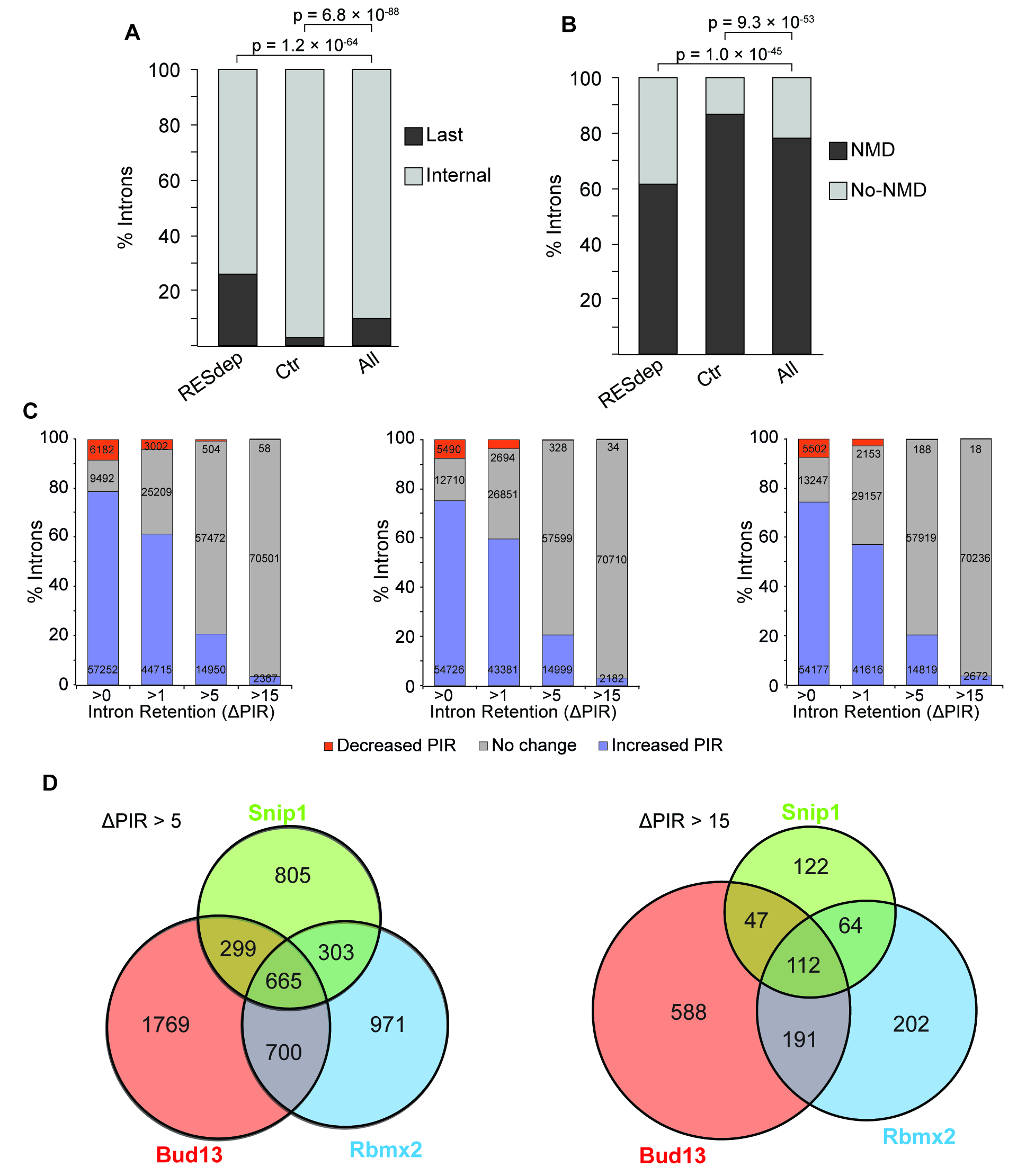

Supplement: S7 Fig — Stacked bar plot showing enrichment for last introns (A) and introns predicted not to trigger NMD upon inclusion (B) among the RES-dependent (RES-dep) introns. On the contrary, introns that are not affected by RES depletion (Ctr) are depleted for these types of introns. P-values were calculated using Fisher exact test. (C) Stacked barplots showing the percentage of introns affected by bud13, rbmx2 or snip1 mutation using different ∆PIR cutoffs. (D) Euler diagram showing the overlap of skkiped exons (∆PIR>5 and ∆PIR>15) affected by bud13, rbmx2, and snip1 mutants. (TIF) [file pgen.1007473.s014.tif]

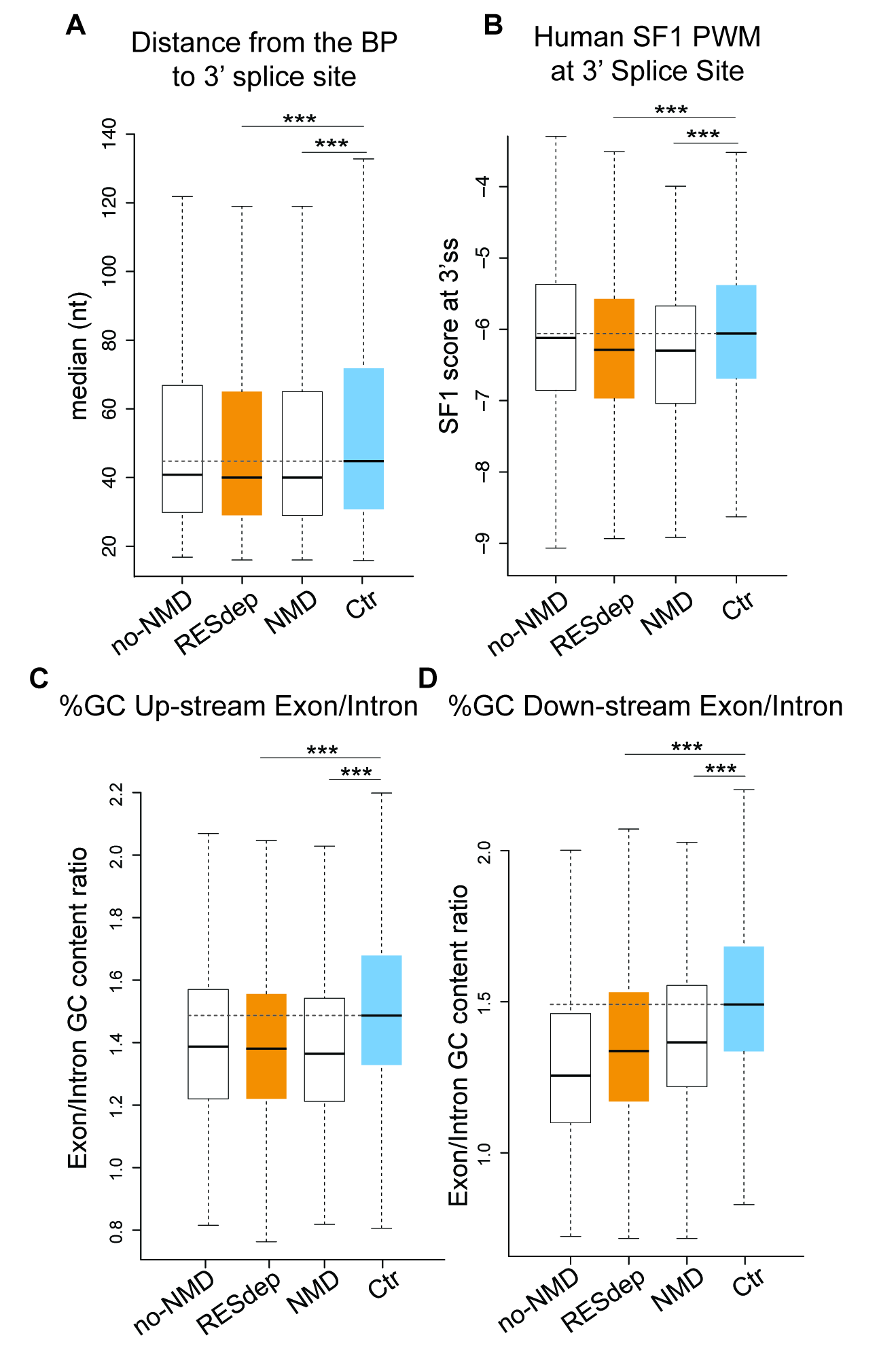

Supplement: S8 Fig — (A) Boxplots showing the distribution of the median nucleotide (nt) distance from the top 3 predicted branch points (BP) to the 3’ splice site for each intron category. (B) Boxplots of the highest score for the human SF1 position weight matrix (PWM) in the 3’ intronic region (see Methods for details). (C-D) Boxplots showing the GC content ratio between up-stream (C) or down-stream (D) exons vs the retained introns. The lower ratio of no- NMD introns, which are enriched in last introns, is caused by the generally low GC content of last exons overlapping the 3' UTR. (***P ≤ 0.001, Mann-Whitney-U test). (TIF) [file pgen.1007473.s015.tif]

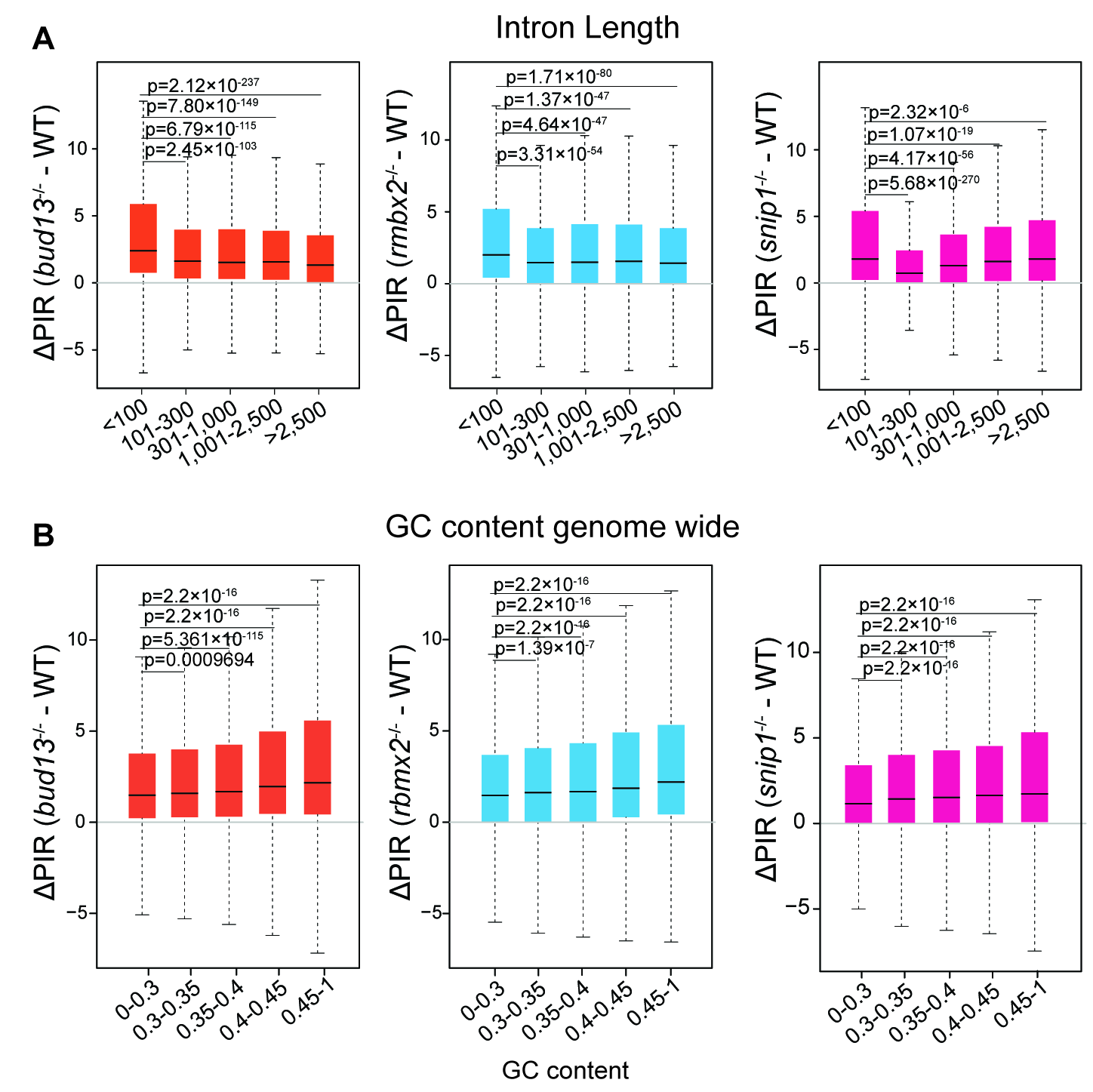

Supplement: S9 Fig — (A) Boxplots showing the degree of change in intron retention (∆PIR) according to intron length. Number of introns per nucleotide bin: ≤100 = 13,059; 101–300 = 13,332; 301–1,000 = 11,808; 1,001–2500 = 18,164; >2500 = 16,563. (B) Boxplots illustrating the degree of change in intron retention (∆PIR) according to intron GC content. Number of introns per bin: 0–0.3 = 14663; 0.3–0.35 = 27361; 0.35–0.4 = 21873; 0.4–0.45 = 6721; 0.45–1 = 2308. P-values were calculated using Wilcoxon rank-sum test. (TIF) [file pgen.1007473.s016.tif]

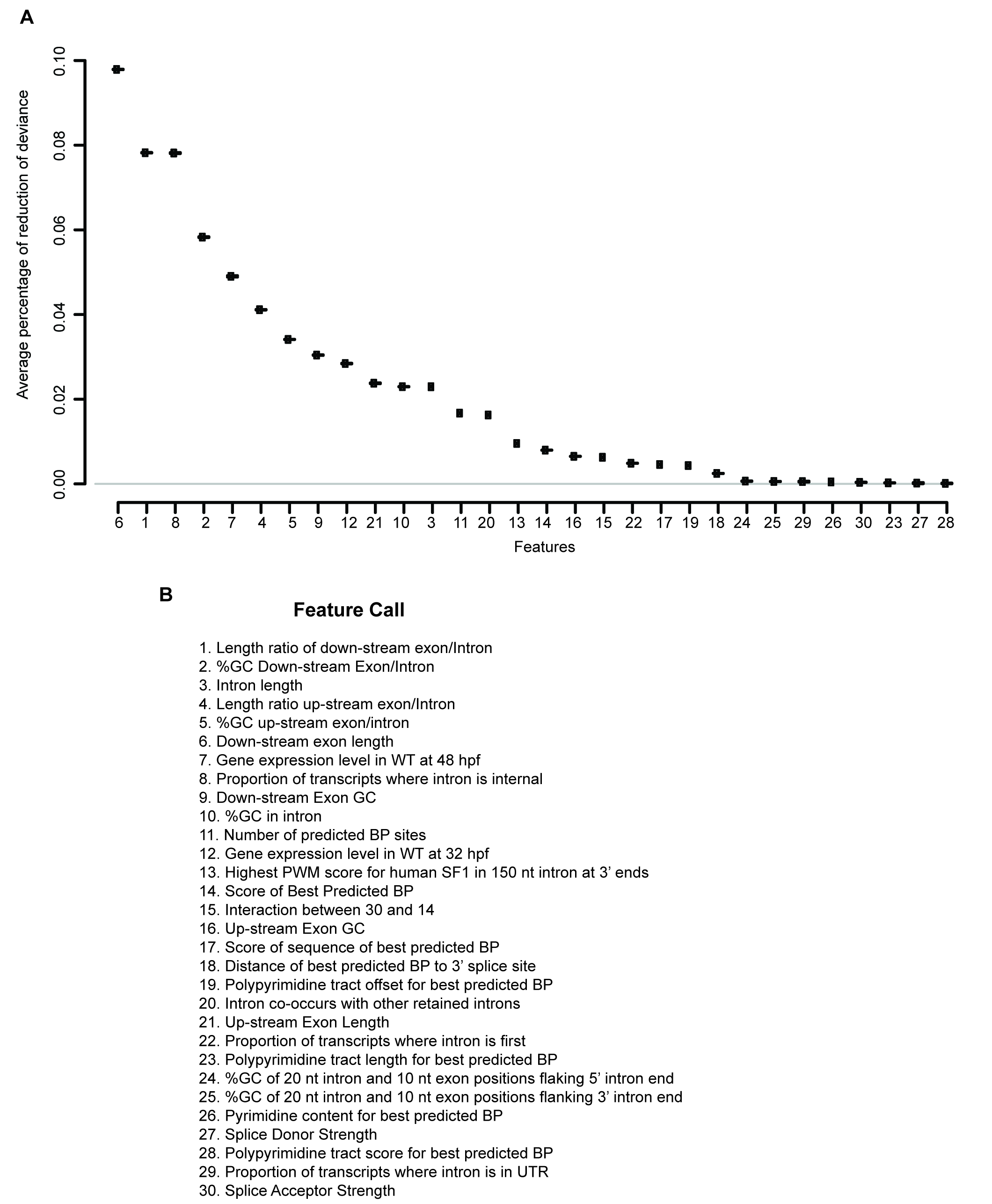

Supplement: S10 Fig — (A) Logistic regression models were learned to discriminate between RESdep and Ctr introns with each feature individually and the fraction of the null deviance that was reduced was recorded. Values were averaged over 10,000 repeated holdout experiments. Training data sets consisted of 1,268 RESdep and 1,268 Ctr introns. Error bars indicate 95% confidence interval of reported averages. (B) Features call used in Fig 6B and in S10A Fig. (TIF) [file pgen.1007473.s017.tif]

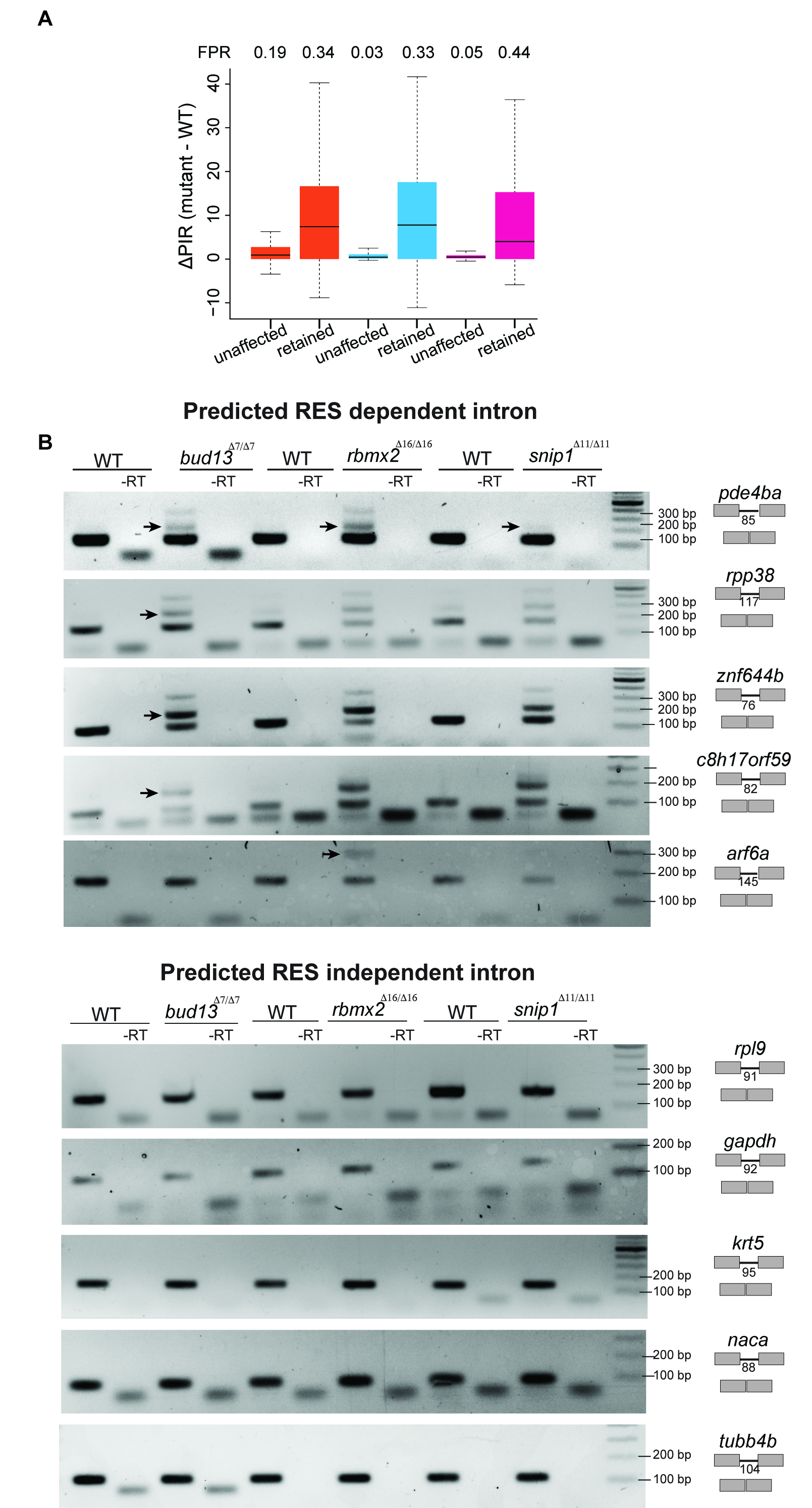

Supplement: S11 Fig — (A) ∆PIR values for the top 100 introns based on their likelihood to be bud13-dependent and -independent, as predicted by the regression model. Only introns with no read coverage across the six RNA-seq samples were used for this analysis (108,470 introns). False positive rate (FPR) values are defined for retained introns to be the fraction of introns with ∆PIR<2, and for unaffected introns to be those with ∆PIR>5. (B) RT-PCR assays validating the predicted RES dependent and RES independent introns in bud13, rbmx2 and snip1 mutants compared with the corresponding phenotypically WT siblings (lower panel). Arrows indicate the retained intron. In the case of arf6a we could detect intron retention by RT-PCR only in the rbmx2 mutant. Unspecific upper band in pde4ba, rpp38, znf644b and c8h17orf59 are likely DNA heteroduplexes. The numbers underneath the introns in the cartoons represent their lengths in bp. (TIF) [file pgen.1007473.s018.tif]

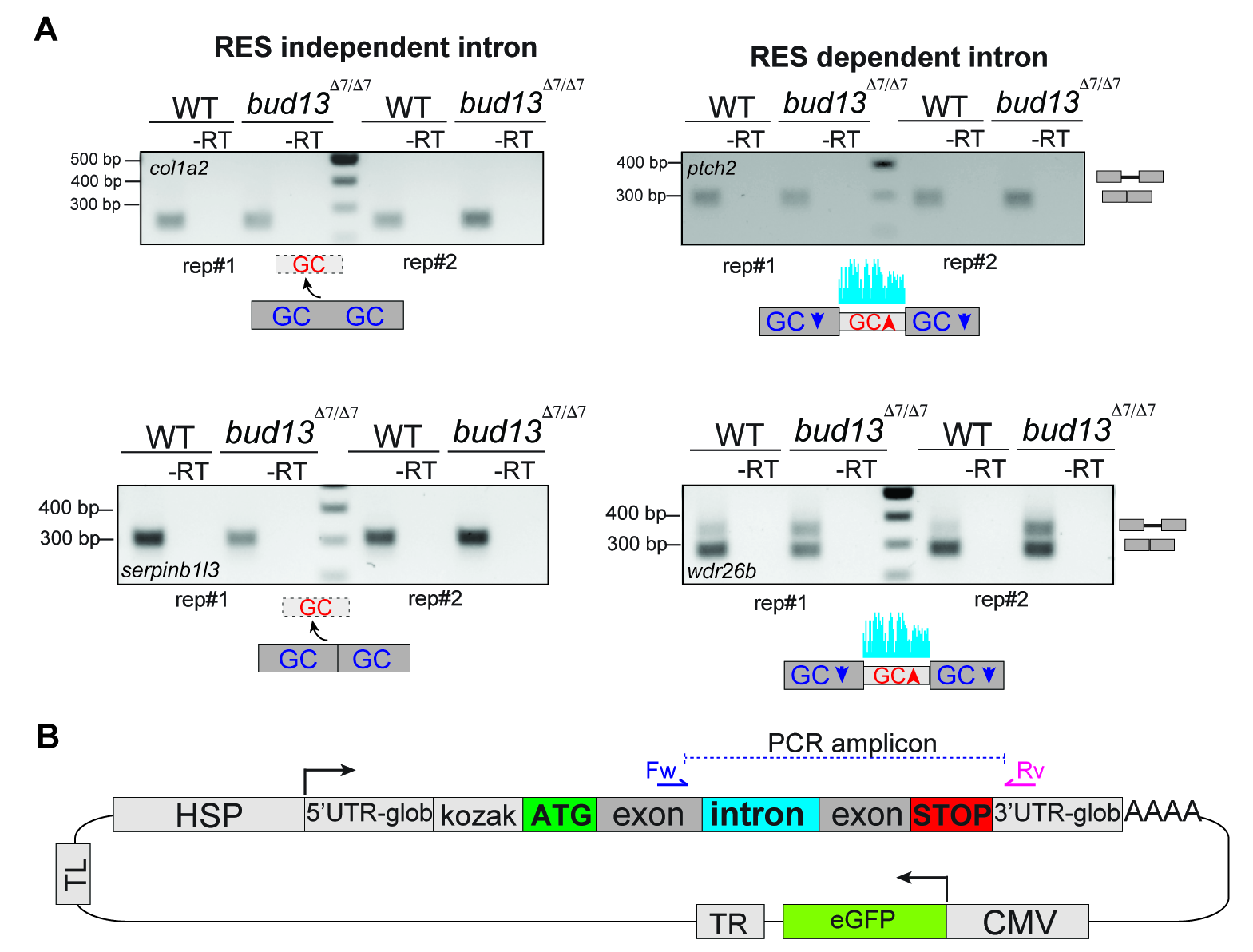

Supplement: S12 Fig — (A) Intron predicted as RES independent were spliced in bud13∆7/∆7 (col1a2 and serpinb1l3) while RES dependent intron wdr26b, but not ptch2, was retained as predicted for the logistic regression model. (B) Scheme of the vector used in the validation assay. PCR product and primers used are depicted as specific Fw primer (blue arrow) and universal Rv primer (magenta arrow). All experiments were done using two independent biological replicates (rep#1 and rep#2). (TIF) [file pgen.1007473.s019.tif]
